# Evolutionary Dynamics of Rift Valley Fever Virus in Saudi Arabia: Whole-Genome Analysis of the 2000–2001 Outbreak Strains

**DOI:** 10.3390/v18101067

**Published:** 2026-09-26

**Authors:** Mohamed A. Farrag, Reem M. Aljowaie, Ibrahim M. Aziz, Abdulaziz Abdullah Almosa, Basel Mohammed Alnafjan, Najat A. Y. Marraiki

**Affiliations:** 1 Department of Botany and Microbiology, College of Science, King Saud University, Riyadh 11451, Saudi Arabia; raljowaie@ksu.edu.sa (R.M.A.); iaziz@ksu.edu.sa (I.M.A.); najat@ksu.edu.sa (N.A.Y.M.); 2 Wellness Prevention Medicine, King Abdulaziz City for Science and Technology (KACST), Riyadh 11451, Saudi Arabia; azimousa@kacst.gov.sa (A.A.A.); bnafjan@kacst.gov.sa (B.M.A.)

**Keywords:** Rift Valley fever virus, Saudi Arabia, phylogenomics, molecular dating, glycosylation, selection pressure, lineage C, reassortment

## Abstract

Rift Valley fever virus (RVFV) is a mosquito-borne zoonotic pathogen that causes severe disease in livestock and humans, with epidemic potential and a capacity for geographic expansion beyond its African endemic range. The 2000–2001 outbreak in Saudi Arabia represented the first confirmed introduction of RVFV outside Africa, yet comprehensive multi-segment genomic characterization of the circulating strains has remained incomplete. Here, we performed an integrated evolutionary and molecular analysis of the three complete-genome Saudi RVFV strains (Saudi 2000-10911, Saudi 200010901, and SA01-1322) using publicly available sequences. Whole-genome comparisons revealed substantial nucleotide and amino acid diversity, with the L segment exhibiting the highest mutation burden (182 nucleotide substitutions, 17 amino acid changes), while the NSs region of the S segment accumulated 52 nucleotide and 11 amino acid mutations compared to the highly conserved nucleocapsid domain. Maximum-likelihood phylogenetic analyses of all three segments consistently placed the Saudi strains within Lineage C (bootstrap = 100/100), clustering with East African strains from Kenya (2006–2007), Tanzania (2007), and South Africa (2008–2010), with congruent topologies across segments confirming the absence of reassortment. Bayesian phylogeographic analysis of 118 L-segment sequences estimated a mean substitution rate of 1.697 × 10^−4^ substitutions/site/year (95% HPD: 1.5–1.9 × 10^−4^) and a time to the most recent common ancestor of approximately 1857 (95% HPD: 1835–1876). Selection-pressure analyses using four complementary methods (MEME, FEL, FUBAR, and SLAC) demonstrated pervasive purifying selection across all three segments, with only sporadic episodic positive selection detected at a limited number of codons (six in L, seven in M, and one in S). In silico glycosylation prediction identified conserved N- and O-glycosylation sites on the M segment, with the Saudi strains displaying a Lineage C-typical profile lacking geographically restricted markers. However, the absence of canonical signal peptides in all sequences warrants cautious interpretation of these predictions. Collectively, our findings are consistent with a single introduction of Lineage C RVFV from East Africa among the sampled strains, with no subsequent reassortment, and that the virus has evolved under strong functional constraints with only rare, lineage-restricted episodes of positive selection. The identified Saudi-specific and lineage-defining mutations provide molecular markers for surveillance, while the high conservation of core proteins supports the feasibility of broadly protective vaccines.

## 1. Introduction

Rift Valley fever is a mosquito-borne zoonotic disease caused by RVFV, a member of the genus *Phlebovirus* within the family *Phenuiviridae*. RVFV was first identified during a 1930–1931 epizootic among sheep in Kenya’s Rift Valley [1]. Subsequently, the virus has spread across sub-Saharan Africa and Madagascar and remains a priority pathogen under the World Health Organization R&D Blueprint because of its epidemic potential and capacity for geographic expansion [2]. The virus primarily affects domestic ruminants, particularly sheep, goats, cattle, and camels, producing high rates of abortion in pregnant animals and elevated mortality in neonates. In humans, most infections are mild or asymptomatic; however, a minority progress to severe disease characterized by hemorrhagic fever, hepatitis, encephalitis, retinitis, or multi-organ failure, with case-fatality rates of 1–15% depending on outbreak severity and clinical presentation [3,4]. The virus is maintained through a dual transmission cycle involving transovarial persistence in *Aedes* mosquitoes and amplification in vertebrate hosts during periods of exceptional rainfall and flooding. Epizootics are strongly associated with El Niño–Southern Oscillation-driven precipitation anomalies that create temporary mosquito breeding habitats [5]. Human infection occurs mainly through direct contact with infected animal tissues or via the bite of infected mosquitoes. Long-distance dispersal is facilitated by livestock trade and, potentially, wind-borne mosquitoes, enabling emergence beyond traditional endemic zones [6,7,8].

The 2000–2001 outbreak in southwestern Saudi Arabia and Yemen represented the first confirmed introduction of RVFV outside the African continent and Madagascar. Triggered by heavy rainfall and flooding in the Jazan region near the Yemeni border, the epidemic produced approximately 886 reported human cases in Saudi Arabia (≈82% laboratory-confirmed) with a case-fatality rate of ≈13.9% (≈123–124 deaths) and an estimated 1087 cases with 121 deaths in Yemen [9,10]. Concurrent livestock losses were substantial. Molecular analyses of partial genomic segments demonstrated that the Arabian isolates were nearly identical to strains circulating during the large 1997–1998 East African epidemic (Kenya, Somalia, Tanzania), supporting a single recent introduction from East Africa, most likely via livestock movement [11,12]. Early multi-segment comparisons indicated congruent phylogenies and no evidence of reassortment among the Saudi and Yemeni isolates [12]. Subsequent complete-genome sequencing of selected Saudi strains (including SA01-1322) confirmed their close relationship to contemporaneous East African viruses [13].

The RVFV genome (≈11.4–12 kb) comprises three single-stranded RNA segments of negative or ambisense polarity. The L segment (≈6.4 kb) encodes the RNA-dependent RNA polymerase; the M segment (≈3.8–3.9 kb) encodes a polyprotein processed into the envelope glycoproteins Gn and Gc, the non-structural protein NSm, and a 78-kDa protein; and the S segment (≈1.7 kb) uses an ambisense strategy to express the nucleoprotein N and the major virulence factor NSs, an interferon antagonist [14,15,16]. Despite overall low nucleotide diversity (typically <5%), phylogenetic analyses of the Gn gene and complete genomes have resolved 15 major lineages (A–O) [15,17]. Lineage C has emerged as particularly successful, originating in southern Africa in the mid-1970s and subsequently expanding across eastern and southern Africa, with documented introductions into Madagascar, the Arabian Peninsula, and more recently Rwanda and other East African countries [18,19,20]. Recent phylodynamic studies estimate substitution rates for lineage C of approximately 3.6–9.8 × 10^−4^ substitutions/site/year across segments and highlight ongoing diversification during inter-epidemic periods [18].

Although early genetic studies established the East African origin and relative genetic homogeneity of the 2000–2001 Saudi isolates and demonstrated congruent phylogenies with no evidence of reassortment among the Saudi and Yemeni strains, comprehensive multi-segment analyses that integrate nucleotide and amino-acid variation, precise lineage assignment, detection of reassortment, Bayesian molecular dating, selection-pressure inference, and glycosylation-site prediction remain limited. Glycosylation of the surface glycoproteins can modulate antigenicity, receptor interactions, and immune evasion, yet its patterns and conservation across lineages have received limited systematic attention. Likewise, signatures of episodic or pervasive positive selection across the three genomic segments of Arabian isolates have not been fully characterized in a global comparative framework. The present study was therefore undertaken to provide an integrated evolutionary and molecular characterization of the three complete-genome Saudi RVFV strains isolated during the 2000–2001 outbreak (Saudi 2000-10911, Saudi 200010901, and SA01-1322). Using publicly available whole-genome sequences of the L, M, and S segments, the study addressed four complementary objectives: (i) detailed nucleotide and amino-acid sequence analyses to identify Saudi-specific, lineage-defining, and globally fixed mutations, combined with in silico prediction of N- and O-glycosylation sites of the segment M glycoproteins; (ii) maximum-likelihood phylogenetic reconstruction and lineage assignment (primarily via the M segment according to the A–O classification system), with comparative trees of the S and L segments to evaluate potential reassortment; (iii) Bayesian molecular dating and phylodynamic inference on an expanded L-segment dataset spanning 1951–2021; and (iv) selection-pressure analysis of the complete coding sequences of all three segments using four complementary methods (MEME, FEL, SLAC, and FUBAR). By placing the Saudi isolates within a robust global genomic context, this work aims to refine understanding of the evolutionary trajectory of the virus responsible for the first extra-African RVF outbreak, clarify its relationships to subsequent East African and other lineages, and identify molecular features of potential adaptive or epidemiological significance. The principal novel contributions of the present study are the integrated whole-genome comparisons across all three segments, the expanded Bayesian dating analysis, the multi-method selection-pressure assessment, and the systematic glycosylation profiling.

## 2. Materials and Methods

### 2.1. Study Design and Data Sources

This in silico study was designed to investigate the evolutionary dynamics, phylogenetic relationships, and molecular characteristics of RVFV strains isolated during the 2000–2001 outbreak in the Kingdom of Saudi Arabia, using publicly available complete whole genome sequences comprising the three genomic segments: Large (L; ~6.4 kb, encoding the RNA-dependent RNA polymerase), Medium (M; ~3.8 kb, encoding the Gn and Gc glycoproteins), and Small (S; ~1.7 kb, encoding the nucleoprotein N and the non-structural protein NSs), as illustrated in Figure 1. The study was structured to achieve four main objectives: (i) Comprehensive nucleotide and amino acid sequence analyses were carried out across all three segments to identify nucleotide mutations and their corresponding amino-acid substitutions, with mutations categorized as Saudi-specific, lineage-defining, or globally fixed; additionally, in silico prediction of N- and O-glycosylation sites was performed on the glycoprotein coding regions of the M segment (Gn and Gc) to assess potential post-translational modifications and their implications for viral fitness and host adaptation. (ii) Phylogenetic analysis and genotype assignment were performed using the M segment as the primary target for lineage classification according to the established major RVFV lineages (A–O), while phylogenetic trees for the S and L segments were reconstructed and compared to the M segment tree to investigate potential reassortment events. (iii) Bayesian molecular dating and phylodynamic inference were conducted on the L segment dataset, comprising 118 sequences (6279 nucleotides) sampled between 1951 and 2021. (iv) Selection pressure analysis was performed on the complete coding sequences of all three segments using four complementary methods—MEME, FEL, SLAC, and FUBAR—to detect signatures of both episodic and pervasive positive selection. The three Saudi strains included in all analyses were (i) Saudi 2000-10911 [DQ380170 (Seg S)/DQ380197 (Seg M)/DQ375401 (Seg L)], (ii) Saudi 200010901 [KU978775 (Seg S)/KU978778 (Seg M)/KU978781 (Seg L)], and (iii) SA01-1322 [KX096943 (Seg S)/KX096942 (Seg M)/KX096941 (Seg L)] with corresponding international reference sequences representing all major RVFV lineages retrieved from the NCBI GenBank database to provide a comprehensive global context. A summary of the sequence datasets used in this study, including the number of sequences, genomic segments, sampling periods and analytical purposes, is provided in Table 1. Accession numbers, strain names, countries of origin and collection years are listed in Supplementary Table S1 (61 complete genomes) and Supplementary Table S2 (118 L-segment sequences).

### 2.2. Sequence Data Retrieval and Processing

Whole genome sequences of RVFV were retrieved from the National Center for Biotechnology Information (NCBI) GenBank database. The search terms applied were “Rift Valley fever virus segment L AND 6404[SLEN]”, “Rift Valley fever virus segment M AND 3885[SLEN]”, and “Rift Valley fever virus segment S AND 1520:1690[SLEN]” for L, M, and S segments, respectively. Sequences with valid sampling or collection dating from 1944 to 2022 were included. To enable robust comparative analysis across all three segments, only strains with complete whole genome sequences available for all three segments were retained, resulting in a unified dataset of 61 strains representing all major RVFV lineages. Sequence datasets were curated by excluding duplicate entries, short fragments, and sequences with high proportions of ambiguous bases. Specifically, the following filtering criteria were applied: (i) duplicate entries were defined as sequences with 100% nucleotide identity and identical metadata (country and year); (ii) short fragments were defined as sequences shorter than the full-length segment; and (iii) sequences with ≥1% ambiguous bases (Ns) were excluded. Retrieved sequences were edited and assembled using BioEdit software (version 7.0). Multiple sequence alignments for each segment were initially performed via the Clustal W algorithm implemented in the MegAlign program (DNASTAR Lasergene, Madison, USA) [version 7.1.0]. The alignments were manually inspected and adjusted as needed using AliView (version 1.28) (Uppsala, Sweden). All whole genome sequences used in this study are publicly available in GenBank; accession numbers are listed in the Supplementary Materials (Table S1). The final unified dataset of 61 high-quality whole genome sequences per segment was used for all downstream analyses.

### 2.3. Nucleotide and Amino Acid Sequence Analysis

Complete genome sequences of the Saudi RVFV strains (segments L, M and S) were analyzed to determine pairwise nucleotide and amino-acid percent identities as well as genetic divergence. Saudi sequences were compared against lineage-specific international reference strains retrieved from GenBank, using the respective prototype strains as consensus sequences. For the S segment, which uses an ambisense coding strategy, the N and NSs open reading frames are encoded in opposite orientations. In the sequence records used, N is annotated in the positive-sense orientation, while NSs is annotated on the complementary strand. To allow simultaneous nucleotide and amino-acid comparisons of both proteins, the NSs open reading frame of every strain was reverse-complemented to the positive-sense coding strand. The resulting positive-sense NSs sequences were then concatenated with the N open reading frame to produce a single contiguous 512-residue alignment (NSs 1–266; N 267–512) that was used for all subsequent mutational and comparative analyses of the S segment. This concatenation was performed solely to provide a uniform alignment coordinate system; it does not imply that NSs and N are expressed as a single protein.

Nucleotide mutations and the resulting amino-acid substitutions were identified across all three segments and categorized as permanent (shared across all lineages except lineage A), lineage-specific, or potentially Saudi-unique. Potential N- and O-glycosylation sites within the glycoprotein coding regions of the M segment (Gn and Gc) were predicted using the NetNGlyc 1.0 server (https://services.healthtech.dtu.dk/services/NetNGlyc-1.0/, accessed on 10 May 2026) and the NetOGlyc 4.0 server, respectively, both with default thresholds (0.5). Only residues scoring above the respective (https://services.healthtech.dtu.dk/services/NetOGlyc-4.0/, accessed on 15 May 2026) thresholds were considered positive. For in silico glycosylation prediction, a subset of 43 sequences was selected from the unified dataset to represent all major RVFV lineages. Conserved N-glycosylation sites were defined as those present in all analyzed sequences, while O-glycosylation patterns were compared across isolates to identify lineage- or geographically associated differences.

### 2.4. Lineage Assignment Using the RVFV Typing Tool

To confirm lineage assignments, the RVFV Typing Tool, a computational method for rapidly classifying and assigning lineages of RVFV isolates, was utilized. The tool is available as a web application at https://www.genomedetective.com/app/typingtool/rvfv/, accessed on 5 May 2026. The glycoprotein (Gn) nucleotide sequences extracted from the M segment of the Saudi strains were submitted to the tool for lineage assignment. The RVFV Typing Tool has been validated on a large dataset and demonstrated 100% specificity, sensitivity, and accuracy in classifying RVFV sequences at the species and phylogenetic levels.

### 2.5. Phylogenetic Analysis

Phylogenetic reconstruction was performed separately on the final unified datasets of whole genome sequences for the L, M, and S segments. The best-fit nucleotide substitution model for each segment was determined using ModelFinder (implemented in IQ-TREE), which selected the General Time Reversible with Invariant sites and Gamma distribution (GTR+I+G4) model for all three segments based on the Bayesian Information Criterion (BIC). Maximum likelihood phylogenetic trees were reconstructed using IQ-TREE version 3.1.3 with 1000 ultrafast bootstrap replicates (UFBoot2) and 1000 SH-aLRT branch tests under the GTR+I+G4 model. The resulting consensus tree files were visualized and annotated using FigTree version 1.4.4. Bootstrap support values ≥ 70% were considered as supporting robust phylogenetic relationships. All phylogenetic trees were rooted at the midpoint.

The M segment tree was reconstructed as the primary phylogenetic analysis, as the M segment encodes the Gn and Gc glycoproteins, which are the primary targets for lineage classification. The M segment tree was used to determine the lineage assignment of the three Saudi strains. To investigate potential reassortment events and confirm lineage consistency, phylogenetic trees were also reconstructed for the S and L segments using the same set of 61 whole genome sequences (Table S1). The S and L segment trees were compared to the M segment tree to detect any incongruences in topology that would indicate reassortment events. Lineage assignment for the S and L segments was performed based on the established 15-lineage classification system (A–O) with reference strains representing each lineage included in the analysis.

### 2.6. Bayesian Molecular Dating and Phylogenetic Inference

A total of 118 L segment sequences (6279 nts) sampled between 1951 and 2021 were aligned using MUSCLE implemented in MEGA11 [21], with the alignment manually inspected and edited in AliView [22]. To assess temporal signal prior to Bayesian analysis, a maximum likelihood tree was inferred, and root-to-tip genetic distances were plotted against sampling dates using TempEst v1.5.4 (Oxford, UK) [23], revealing a strong temporal structure (correlation coefficient = 0.9057, R^2^ = 0.8203, slope = 3.1073 × 10^−4^ substitutions/site/year, residual mean square = 5.71 × 10^−6^), with the x-intercept estimated at approximately 1908.5. Bayesian phylogenetic analyses were conducted in BEAST X (v10.5.0) [24] via the BEAUti interface, employing the GTR nucleotide substitution model [25] (without gamma-distributed rate variation).

Both strict and uncorrelated lognormal relaxed molecular clocks, as well as constant-size and Bayesian skyline coalescent tree priors, were evaluated. The relaxed-clock models consistently showed poor mixing and low effective sample size (ESS) values for several key parameters. In contrast, the combination of a strict molecular clock and a constant-size coalescent prior provided optimal mixing and high ESS values (>400) for all major parameters and was therefore selected for the final analysis. This choice is further supported by the strong linear temporal signal observed in TempEst, which is consistent with a relatively constant evolutionary rate across the sampled L-segment dataset.

A sequence file was prepared and imported into BEAUti, where sampling dates were incorporated as tip dates to generate the XML input file. The Markov chain Monte Carlo (MCMC) was run for 100 million generations, sampling every 2000 states. To achieve efficient exploration of the posterior distribution and minimize autocorrelation, MCMC operators were extensively tuned (e.g., upDownOperator weight = 50; subtreeLeap size = 30.0). Convergence was rigorously assessed using Tracer v1.7.2 (Edinburgh, UK), with all key parameters achieving Effective Sample Sizes (ESS) well above 400 (e.g., treeLength ESS = 455; clock.rate ESS = 501; age(root) ESS = 539). A Maximum Clade Credibility (MCC) tree was generated from the post-burn-in trees using TreeAnnotator v10.5.0 after discarding the first 10% of generations as burn-in, with node heights summarized as medians. Final tree was visualized and annotated in FigTree v1.4.4, and root heights (in substitutions/site) were converted to divergence times (calendar years) using the time-calibrated MCC trees.

### 2.7. Selection-Pressure Analysis

Complete coding sequences of the Saudi RVFV strains (segments L, M and S) were aligned at the codon level using the MUSCLE algorithm implemented in MEGA11 and subsequently inspected and edited in AliView. To obtain an in-frame codon alignment, the protein alignment was generated and back-translated to the corresponding codon alignment using the kc-align tool (v.1.0.2+galaxy1) within the Galaxy platform [26]. kc-align is a codon-aware multiple aligner that uses Kalign3 to produce in-frame gapped codon alignments suitable for selection analysis. The resulting codon alignments, comprising the Saudi strains plus lineage-specific background sequences, were subjected to positive-selection analysis using the Datamonkey 2.0 web server [27], which implements the HyPhy package [28]. Four complementary methods were employed: MEME [29] to detect episodic diversifying selection, and FEL [28], SLAC [30] and FUBAR [29] to detect pervasive selection. A site was reported as under episodic positive selection if it reached *p* ≤ 0.05 in MEME. Sites under pervasive positive selection were evaluated with the conventional thresholds of *p* ≤ 0.05 (FEL, SLAC) or posterior probability ≥ 0.9 (FUBAR). Because the three pervasive-selection methods recovered no sites meeting these thresholds, only the MEME results are presented as evidence of positive selection.

## 3. Results

### 3.1. Nucleotide and Amino Acid Variability Across the Three Genomic Segments

Whole-genome sequence analysis of Saudi RVFV strains demonstrated considerable nucleotide and amino-acid diversity across all three genomic segments. In the M segment, 114 nucleotide substitutions (114/3594) resulted in 12 amino-acid changes (I60V, D95N, F118G, A123T, L232Q, Y433F, D566G, I631V, S1059T, N662D, V685I, D987E), four of which (F118G, A123T, Y433F, N662D) were unique to the Saudi isolates; several residues (L232Q, D566G, I631V, S1059T) were fixed in all lineages except lineage A, while additional lineage-restricted substitutions were observed (I863V in lineage O; I60V and D95N in lineage C; T1130I in lineage H) (Figure 2A and Figures S1–S11). The L segment exhibited the highest variability, with 182 nucleotide mutations (182/6279), of which 17 changed their corresponding amino acids. Saudi strains had six specific changes (V164I, F291L, S411G, T577A, S1656N, S1670P) and multiple lineage-defining polymorphisms (R244K/A426G in lineage O; R249K/V349I/D446N in lineage C; V336A in lineage N; M120T/E177D/N270S/H1852Y/R1926K in lineage H; M406V in lineage K). Five amino acid residues, F23Y, S278N, S470N, A663T, D1984N, are conserved among all lineages except lineage A (Figure 2B and Figures S12–S31). Within the S segment, the non-structural protein coding region (residues 1–266) accumulated 52 nucleotide and 11 amino-acid mutations (F23I, R24K, V62A, A167V, V189I, V217A, I239V, I242V, M250I, E253G, V262A), five of which were exclusive to Saudi strains, whereas four substitutions (F23I, A167V, V217A, I242V) were permanent across lineages other than A; the nucleocapsid protein domain (residues 267–512) was markedly more conserved, harbouring only the single fixed change G425E outside lineage A (Figure 2C and Figures S32–S36). Collectively, these genome-wide patterns indicate moderate-to-high mutational pressure preferentially affecting the polymerase and non-structural regions, together with a distinct signature of Saudi-specific and lineage-defining substitutions that refine understanding of local RVFV evolution and molecular epidemiology.

### 3.2. Phylogenetic Analysis of the Segment M

Phylogenetic analysis of the M segment was performed on a unified dataset of 61 whole genome sequences, including the three Saudi strains and reference strains representing all major RVFV lineages. The maximum likelihood tree revealed that the three Saudi strains formed a well-supported monophyletic clade within Lineage C (bootstrap = 100/100) (Figure 3A). The Saudi strains clustered most closely with Kenyan strains from 2007 (EU574043, HM586965, HM586966, HM586967), Tanzanian strains from 2007 (HM586970), and South African strains from 2008–2010 (KX944840, KX944842, KY126705, KY126706), suggesting a shared origin with East African outbreaks. The close genetic relationship and the high bootstrap support are consistent with a single introduction event of Lineage C RVFV among the sampled strains into Saudi Arabia during the 2000–2001 outbreak. The M segment tree also resolved the established RVFV lineages, with Lineage A represented by the Egyptian reference strain ZH-548 (NC_014396) and Lineage K represented by the Ugandan strains Lunyo (KU167026, OQ440143) and Entebbe (DQ380191).

### 3.3. Comparative Analysis of the S and L Segments

To investigate potential reassortment events and confirm lineage consistency, phylogenetic analyses were also performed on the S and L segments using the same 61 whole genome sequences. The S segment tree (Figure 3C) showed that the Saudi strains (DQ380170, KU978775, KX096943) formed a well-supported monophyletic clade within Lineage C (bootstrap = 100/100). Similarly, the L segment tree (Figure 3B) placed the Saudi strains (DQ375401, KU978781, KX096941) within Lineage C with high bootstrap support (100/100). Across all three segments, the Saudi strains consistently clustered with the same East African reference strains from Kenya (2006–2007), Tanzania (2007), and South Africa (2008–2010). Notably, the congruent topologies observed across the L, M, and S segments provided no evidence of reassortment among the Saudi strains, as all three segments placed the strains within the same lineage (Lineage C) with bootstrap support values exceeding 94%. The consistent lineage assignment across all three genome segments further supports the conclusion that the 2000–2001 Saudi Arabian outbreak is consistent with a single viral introduction among the sampled strains, likely originating from East Africa, and that the RVFV genome has remained stable in the region without detectable reassortment events.

### 3.4. Spatial-Temporal Dynamics and Phylogeny of RVFV

Bayesian phylogenetic analysis of 118 L-segment sequences (6279 nts) sampled over a 70-year period (1951–2021) was conducted under a GTR substitution model with a strict molecular clock and a constant-size coalescent prior (Figure 4). This produced a well-resolved, time-calibrated phylogeny with all key parameters achieving effective sample sizes well above 400, confirming robust MCMC convergence. The estimated mean substitution rate was 1.697 × 10^−4^ substitutions per site per year (95% HPD: ~1.5–1.9 × 10^−4^). When referenced to the most recent sampling year (2021), this placed the time to the most recent common ancestor (tMRCA) at approximately 1857 (95% HPD: 1835–1876), suggesting that the sampled RVFV diversity emerged in the mid-19th century. The Maximum Clade Credibility (MCC) tree exhibited strong phylogenetic support, with a median posterior clade credibility of 0.975 and 61 internal nodes displaying posterior probabilities exceeding 0.95. The tree recovered distinct lineages whose sampled members are geographically clustered. These include a basal Southern African clade (South Africa, Zimbabwe, Namibia), a highly diverse East African clade (encompassing early Ugandan and Kenyan strains), a well-supported West African lineage, and a monophyletic Saudi Arabian/Asian clade. Notably, the three Saudi Arabian strains isolated in 2000–2001 (DQ375401, KU978781 and KX096941) formed a well-supported cluster within this clade and were closely related to recent outbreak sequences from China (2016) and Angola, alongside contemporaneous circulating strains from Uganda and Kenya (2018–2021). The total tree length was estimated at 1979.8 substitutions, and the constant-size coalescent model estimated an effective population size (Ne) of approximately 136.4, indicating relative genetic stability in the viral population over the evolutionary timescale sampled. These geographic associations reflect the sampling locations of the analyzed strains; no formal spatial diffusion model was implemented.

### 3.5. Prediction of O- and N-Glycosylation Sites of the M Segment Proteins

In silico glycosylation prediction of the M segment proteins was mainly achieved using NetOGlyc 4.0.0.13 and NetNGlyc 1.0. The analysis revealed varying degrees of conservation and strain-specific variation. O-glycosylation analysis of the M segment identified nine sites in total (Table S3, Figures S1–S11). Five of these sites (414, 797, 801, 806 and 980) were highly conserved, occurring in ≥90% of strains. Site 984 was present in 79% of strains, while sites 387 and 400 occurred at intermediate frequencies. Site 178 appeared exclusively in HM586967-Ken-Mal-032-07-KENYA-2007 and KX611606-Beijing-01-China-2016. All three Saudi strains (KU978778, KX096942 and DQ380197) lacked site 984 but retained the highly conserved core (414, 797, 801, 806 and 980), carrying either site 387 (KX096942) or site 400 (KU978778 and DQ380197). N-glycosylation analysis of the M segment revealed six conserved positions (88, 438, 794, 829, 1035, and 1077), with site 517 uniquely observed in DQ380211-73HB1449-Central African Republic-1974 and site 1035 absent only in DQ380185-OS-8-Mauritania-1987. All three Saudi strains (KU978778, KX096942, and DQ380197) harbored the complete set of six conserved N-glycosylation sites, mirroring the majority of global strains and showing no lineage-specific deletions.

### 3.6. Selection Pressure Analysis

Selection-pressure analyses of the complete coding sequences of the L (RdRp; 2092 codons), M (Gn/Gc polyprotein; 1197 codons) and S (N + NSs; 510 codons) segments were performed with four complementary methods implemented on the Datamonkey adaptive-evolution platform: MEME (episodic diversifying selection), FEL (pervasive positive/negative selection), FUBAR (Bayesian pervasive selection) and SLAC (counting-based dN/dS) (Table 2). Across all three genomic segments the predominant selective regime was purifying (negative) selection, consistent with strong functional constraints on essential viral proteins. Under FUBAR, no codon reached the conventional posterior-probability threshold of ≥0.9 for pervasive positive selection; the highest probabilities observed were 0.88 (M-segment codon 987), 0.87 (S-segment codons 133 and 202) and 0.80 (L-segment codon 411). In contrast, a large fraction of sites showed strong evidence of purifying selection (posterior probability of positive selection ≤ 0.1): 1050 sites in the L segment, 581 sites in the M segment and 161 sites in the S segment. FEL corroborated this pattern, detecting 344, 21 and 82 sites under significant purifying selection (*p* ≤ 0.05) in the L, M and S segments, respectively, while recovering no sites under pervasive positive selection at the same significance threshold. SLAC, a more conservative counting-based method, likewise detected no sites under positive selection (P[dN/dS > 1] ≤ 0.05) in any segment and identified 30, 76 and 30 sites under significant purifying selection (P[dN/dS < 1] ≤ 0.05) in the L, M and S segments, respectively, reinforcing the overall pattern of constraint. MEME, which is designed to detect episodic (branch-specific) diversifying selection, identified a modest number of codons that have experienced intermittent diversifying selection (*p* ≤ 0.05). No codon was identified as positively selected by two or more of the four methods; the MEME-only hits are therefore reported as candidate episodic sites. In the L segment six sites (codons 239, 789, 1244, 1245, 1630 and 1728) were identified as candidates under episodic diversifying selection (MEME only). In the M segment seven sites (codons 173, 238, 286, 431, 554, 574 and 631) were identified as candidates under episodic diversifying selection (MEME only). In the S segment only a single site (codon 133) met the significance threshold for MEME. At each of these candidate sites the proportion of branches flagged by MEME was low (typically one or two branches), indicating that diversifying pressure has been restricted to particular lineages or epidemic episodes rather than acting continuously across the phylogeny. Collectively, the four methods demonstrate that the RVFV genomes examined here, including the three Saudi Arabian isolates from the 2000–2001 outbreak, evolve under pervasive purifying selection, with only sporadic, lineage-restricted episodes of candidate episodic diversifying selection (MEME only). These candidate episodic sites, particularly those located in the M-segment glycoproteins, warrant further experimental investigation for possible roles in host adaptation, immune escape or vector competence.

## 4. Discussion

This study provides a detailed genomic characterization of RVFV strains responsible for the 2000–2001 outbreak in Saudi Arabia, revealing substantial nucleotide and amino acid diversity, distinct lineage-defining mutations, consistent phylogenetic placement without reassortment, and a predominantly purifying selective regime. These findings have important implications for understanding RVFV evolution, geographic lineage distribution, glycoprotein functionality, and the design of surveillance and intervention strategies. Whole-genome analysis demonstrated that the L segment exhibited the highest absolute number of mutations (182 nucleotide substitutions, 17 amino acid changes), followed by the M (114 substitutions, 12 changes) and S segments (52 substitutions in the NSs region). Although the L segment is generally considered to evolve more slowly on a per-site basis [20,31], its larger coding capacity inevitably accumulates more total changes over time. This observation aligns with recent genomic surveillance efforts in East Africa, where the polymerase gene often shows higher nucleotide diversity than the glycoprotein or nucleocapsid genes [15,32]. Importantly, several Saudi-specific amino acid replacements—such as F118G, A123T, Y433F and N662D in the M segment, and V164I, F291L, S411G, T577A, S1656N and S1670P in the L segment—were identified. The fact that numerous residues (e.g., L232Q, D566G, I631V, S1059T) were fixed in all lineages except lineage A underscores the evolutionary distinctiveness of the Egyptian-related lineage A, a pattern consistently observed in large-scale phylogenetic studies [11,15,19,33]. The presence of lineage-restricted polymorphisms, such as I863V in lineage O and I60V/D95N in lineage C, further supports the ongoing diversification of RVFV into geographically and ecologically defined clades, as recently mapped across Africa and the Arabian Peninsula [14,16].

Within the S segment, the non-structural protein (NSs) region accumulated 52 nucleotide and 11 amino acid mutations, whereas the nucleocapsid (N) domain was highly conserved, with only the fixed change G425E outside lineage A. This stark contrast is biologically meaningful: NSs is the primary interferon antagonist and virulence determinant, and its higher variability likely reflects immune-driven adaptation, while the N protein is functionally constrained due to its essential role in RNA encapsidation and ribonucleoprotein formation [34,35,36,37]. Recent reviews have emphasized that NSs is a major target of host-species-specific selection, which may explain the rapid evolution observed in this region [36,38]. The high conservation of the N protein, on the other hand, supports its utility as a diagnostic antigen and potential vaccine target, as recently demonstrated in mRNA vaccine platforms [39,40].

Phylogenetic analyses of all three segments consistently placed the Saudi strains within Lineage C, forming a well-supported monophyletic clade (bootstrap = 100/100) with East African strains from Kenya (2006–2007), Tanzania (2007) and South Africa (2008–2010). The congruent topologies across L, M and S provide no evidence of reassortment, which is a notable finding given that segmented viruses often exchange genome segments during co-infections. In RVFV, reassortment has been documented in nature, particularly between lineages circulating in overlapping geographic regions [31,41,42], but the Saudi strains appear to have maintained intact genome constellations. This observation is in line with recent interepidemic genomic surveillance in Kenya and Rwanda, where no reassortment events were detected among RVFV isolates [16,19]. The absence of reassortment among the sampled strains is consistent with the introduction of a single, genetically homogeneous entity, likely originating from East Africa, a hypothesis reinforced by the close genetic relationship between Saudi strains and those from the 2006–2007 East African epizootic [19]. The high bootstrap support and consistent lineage assignment across all segments also confirm that the molecular epidemiology of RVFV in the Arabian Peninsula has been relatively stable since its introduction, without detectable genomic exchanges with other lineages.

The MCC tree recovered distinct geographic lineages, including a Southern African clade, a highly diverse East African clade, and a West African lineage, with Saudi strains clustering within the East African lineage C [14,17]. Saudi strains belong to lineage C, which also includes recent strains from Uganda and Kenya (2018–2021), indicating ongoing connectivity between East Africa and the Arabian Peninsula [14]. This is consistent with recent genomic epidemiology showing that RVFV has been progressively breaking geographical barriers, with human infection rates rising significantly and 60% of outbreaks occurring within 0–2 months after heavy rainfall or flooding [43]. The effective population size (Ne ≈ 136.4) under the constant-size coalescent suggests relative genetic stability, which aligns with the predominantly purifying selection we observed. Nevertheless, the continuous introduction of viruses from East Africa, as recently documented in Rwanda [19], underscores the need for ongoing genomic surveillance to detect potential changes in population dynamics.

In silico glycosylation prediction of the M-segment proteins revealed notable conservation alongside limited strain-specific variation. Several sites were highly conserved across the examined isolates, particularly the O-glycosylation site at position 414 and the six N-glycosylation sites at positions 88, 438, 794, 829, 1035 and 1077. The absolute conservation of these residues across diverse geographic and temporal isolates suggests a fundamental functional role, potentially related to protein folding, structural stability or receptor interactions [44,45]. In contrast, O-glycosylation site 984 was present in only 79% of strains and was absent from all three Saudi isolates (KU978778, KX096942 and DQ380197). These strains otherwise retained the highly conserved core (sites 414, 797, 801, 806 and 980) and carried either site 387 or 400. Such variable sites may reflect adaptive differences driven by host immune pressure or local ecological factors, as previously hypothesized for lineage-specific glycosylation patterns [46]. Overall, the Saudi strains displayed a glycosylation profile typical of Lineage C, reinforcing their phylogenetic placement and East African origin.

Collectively, the Saudi RVFV strains display a glycosylation profile typical of Lineage C, characterized by the presence of core conserved sites on the segment M. This profile robustly reinforces the phylogenetic placement of the Saudi strains within Lineage C and is consistent with a single introduction event from East Africa among the sampled strains [16,19].

Rigorous experimental validation—such as site-directed mutagenesis, lectin-binding assays, and mass spectrometry—is essential to confirm the functional relevance of the identified glycosylation sites and to determine whether they genuinely contribute to viral fitness, immune evasion, or vector competence [44]. Recent reviews continue to emphasize glycoproteins as key virulence factors in RVFV pathogenesis [47], and structural studies on RVFV glycoproteins remain an active area of research [48].

Using four complementary methods (MEME, FEL, FUBAR and SLAC), we demonstrated that purifying (negative) selection predominates across all three genome segments. No codon reached the significance threshold for pervasive positive selection under FUBAR, FEL, or SLAC, and no codon was identified by more than one method. This is consistent with earlier reports that RVFV evolution is highly constrained, likely due to the alternating vertebrate–mosquito transmission cycle, which imposes stabilizing selection on essential viral proteins [32]. However, MEME, which detects episodic (branch-specific) diversifying selection, identified a modest number of candidate sites—six in the L segment, seven in the M segment and one in the S segment—with the signal restricted to one or two branches. Because none of these sites met a two-method concordance criterion, they are reported as candidates for episodic diversifying selection rather than validated positively selected residues. These candidate sites, particularly those in the glycoprotein (M segment), may reflect transient adaptation to new hosts or immune pressures, but require experimental validation. A recent comparative codon usage analysis found that the N protein from viruses in regions with no prior RVF history had higher codon adaptation indices, suggesting adaptive evolution to human hosts [49].

In addition, a hyper-attenuated variant generated by favipiravir revealed potential virulence markers in the polymerase, further supporting the notion that specific residues in the L segment can modulate pathogenicity [50]. Although the overall selective pressure is purifying, the episodic positive selection sites identified here warrant further experimental investigation, as they may contribute to immune escape or vector competence, as recently highlighted in studies on mosquito susceptibility [32]. The reproductive mosquito–lamb–mosquito cycle has been successfully reproduced in laboratory settings, confirming that over 50 mosquito species can transmit RVFV [51], and variation in the glycoproteins may influence vector-specific transmission efficiency.

The high conservation of the nucleocapsid protein and the core glycoprotein regions, despite overall genetic diversity, supports the notion that a single broadly protective vaccine may be effective against all current lineages. This is particularly encouraging given the recent success of a ChAdOx1-vectored RVF vaccine, which has completed Phase I clinical trials in humans and demonstrated protective efficacy in livestock [52]. Moreover, mRNA vaccines encoding Gn and Gc have elicited strong immune responses in mice and rhesus macaques [39], underscoring the translational value of the conserved sequences we identified. The absence of reassortment and the single introduction event into Saudi Arabia also indicate that the virus has not undergone major genomic reshuffling in the region, which simplifies diagnostic and vaccine strategies. However, the increasing frequency of RVFV outbreaks and its spread to new geographic areas, as documented in recent genomic surveillance studies [19], highlight the need for continuous genomic surveillance to monitor for emerging variants, potential reassortment, and changes in glycosylation patterns that could affect antigenicity or vector competence. The identification of Saudi-specific and lineage-defining mutations provides molecular markers that can be rapidly deployed for outbreak source tracking and risk assessment.

Several constraints should be acknowledged. First, the study relies on publicly available sequences; only three Saudi complete genomes were available, limiting the resolution of within-outbreak diversity and the power to detect rare reassortants or low-frequency variants. Second, glycosylation predictions must be interpreted with caution: the absence of canonical signal peptides in all examined sequences implies that the predicted N- and O-linked sites may not be processed by the host secretory pathway in vivo; experimental validation (mass spectrometry, site-directed mutagenesis, lectin assays) is therefore required. Third, selection-pressure inference is sensitive to dataset composition and the statistical thresholds employed; although four independent methods converged on a predominantly purifying regime, additional sequences from inter-epidemic periods and under-sampled African lineages would strengthen the detection of lineage-specific adaptive episodes. Fourth, we did not implement a formal phylogeographic diffusion model (such as discrete trait analysis with BSSVS or continuous phylogeographic reconstruction). The geographic patterns reported here therefore reflect the sampling locations of the analyzed strains and should be interpreted as descriptive associations rather than as a reconstructed spatial diffusion history. Finally, the functional consequences of the identified Saudi-specific and episodically selected residues remain untested; reverse-genetic and experimental-infection studies will be necessary to determine their impact on virulence, vector competence or immune escape. Despite these limitations, the multi-segment phylogenetic congruence, the clear East-African origin, the absence of reassortment, and the strong functional constraints documented here provide a robust genomic framework for ongoing surveillance and vaccine design against RVFV on the Arabian Peninsula and beyond.

## 5. Conclusions

The integrated whole-genome analyses presented here establish that the three complete-genome RVFV strains recovered during the 2000–2001 Saudi Arabian outbreak belong unequivocally to Lineage C and form a tightly supported monophyletic clade with contemporaneous and subsequent East African isolates (Kenya 2006–2007, Tanzania 2007, South Africa 2008–2010). Congruent topologies across the L, M and S segments provide no evidence of reassortment, consistent with the virus having entered the Arabian Peninsula as a single, genetically coherent entity among the sampled strains—most probably via livestock movement—and has remained genomically stable thereafter. Bayesian dating of an expanded L-segment dataset places the most recent common ancestor of the sampled diversity in the mid-nineteenth century (≈1857; 95% HPD 1835–1876) under a mean substitution rate of 1.697 × 10^−4^ substitutions/site/year, consistent with a long evolutionary history punctuated by episodic geographic expansions. Nucleotide and amino-acid variation is unevenly distributed: the polymerase (L) and the interferon-antagonist NSs domain accumulate the greatest mutational load, whereas the nucleoprotein and core glycoprotein regions remain highly conserved. Selection analyses employing four complementary methods (MEME, FEL, FUBAR, SLAC) demonstrate that purifying selection is the dominant evolutionary force across all three segments; candidate episodic diversifying selection (MEME only) is restricted to a small number of codons and limited phylogenetic branches; no codon was validated as positively selected by more than one method. Glycosylation profiling recovers a Lineage-C-typical pattern lacking several geographically restricted markers, reinforcing the East-African origin of the Saudi strains. Collectively, these findings refine the molecular epidemiology of the first extra-African RVF outbreak, supply lineage-defining and Saudi-specific markers useful for genomic surveillance, and underscore the functional conservation of key antigenic domains that supports the feasibility of broadly protective vaccines.

## Figures and Tables

**Figure 1:**
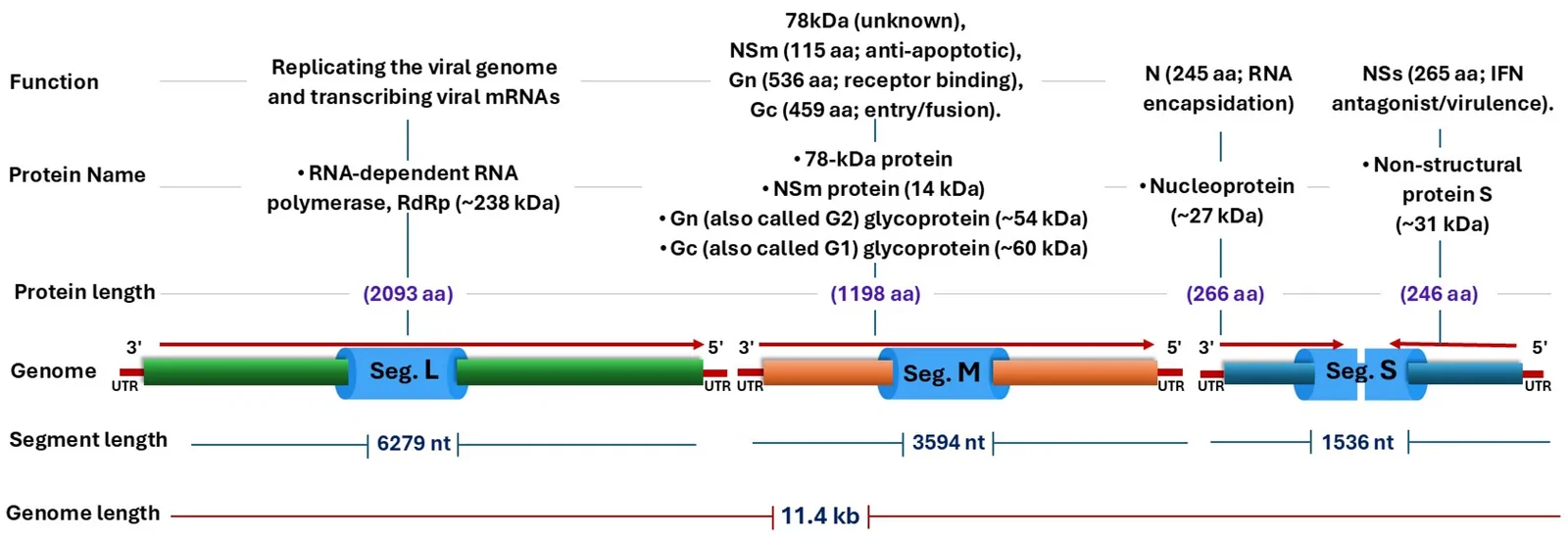
Genomic organization of RVFV. The three genomic segments (L, M, and S) are shown with their respective untranslated regions (UTRs). Segment L (6279 nt) encodes the RNA-dependent RNA polymerase (RdRp; ~238 kDa, 2093 aa), which replicates the viral genome and transcribes viral mRNAs. Segment M (3594 nt) encodes a polyprotein (1198 aa) that is post-translationally cleaved into the 78-kDa protein, NSm (14 kDa; anti-apoptotic), Gn (G2 glycoprotein; ~54 kDa; receptor binding), and Gc (G1 glycoprotein; ~60 kDa; entry/fusion). Segment S (1536 nt) encodes the nucleoprotein N (~27 kDa, 266 aa; RNA encapsidation), generated from an mRNA transcribed from the negative-sense genomic viral RNA, and the non-structural protein NSs (~31 kDa, 246 aa; IFN antagonist/virulence), produced from an mRNA transcribed from the antigenomic copy. The total genome length is 11.4 kb.

**Figure 2:**
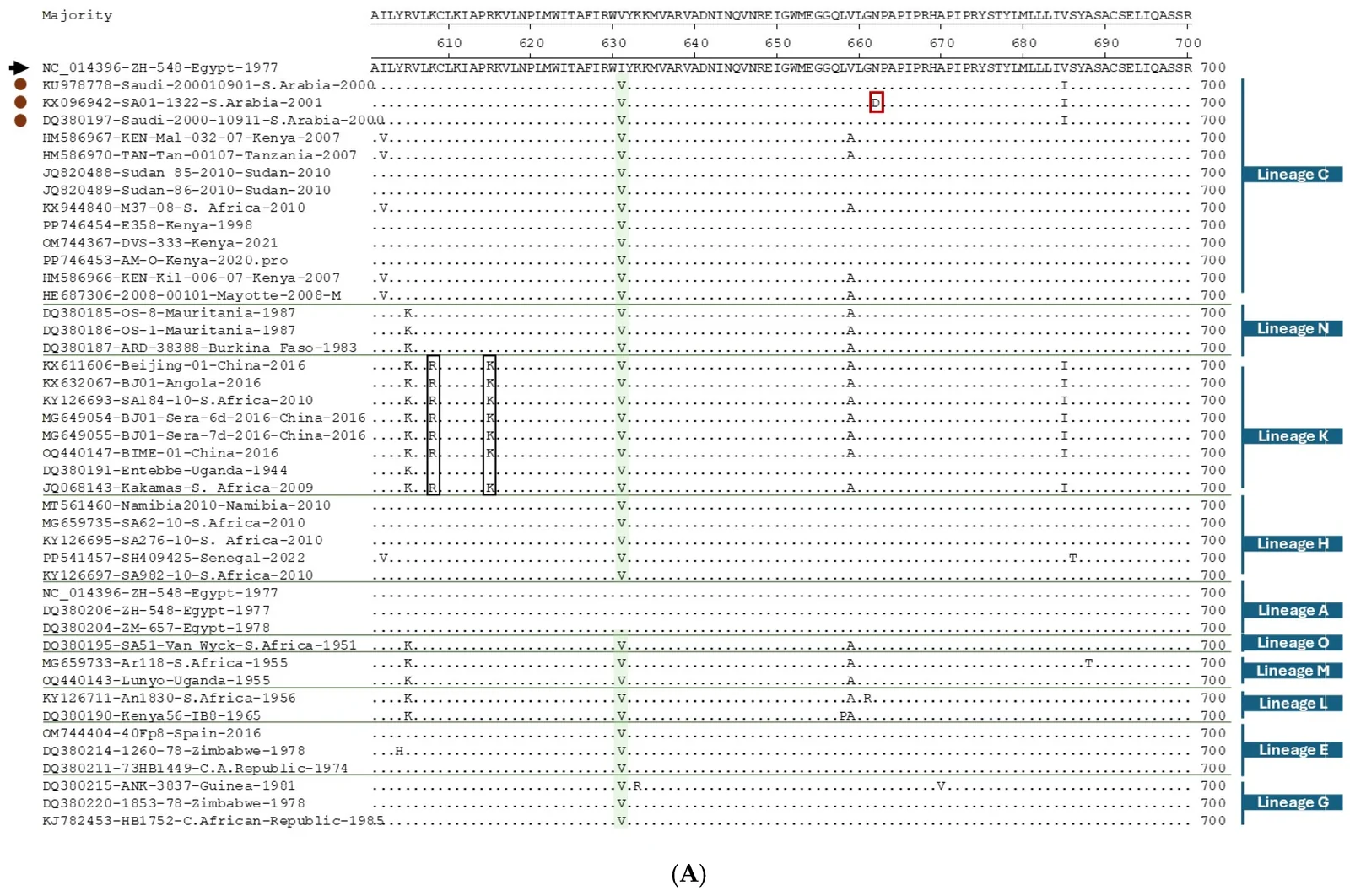

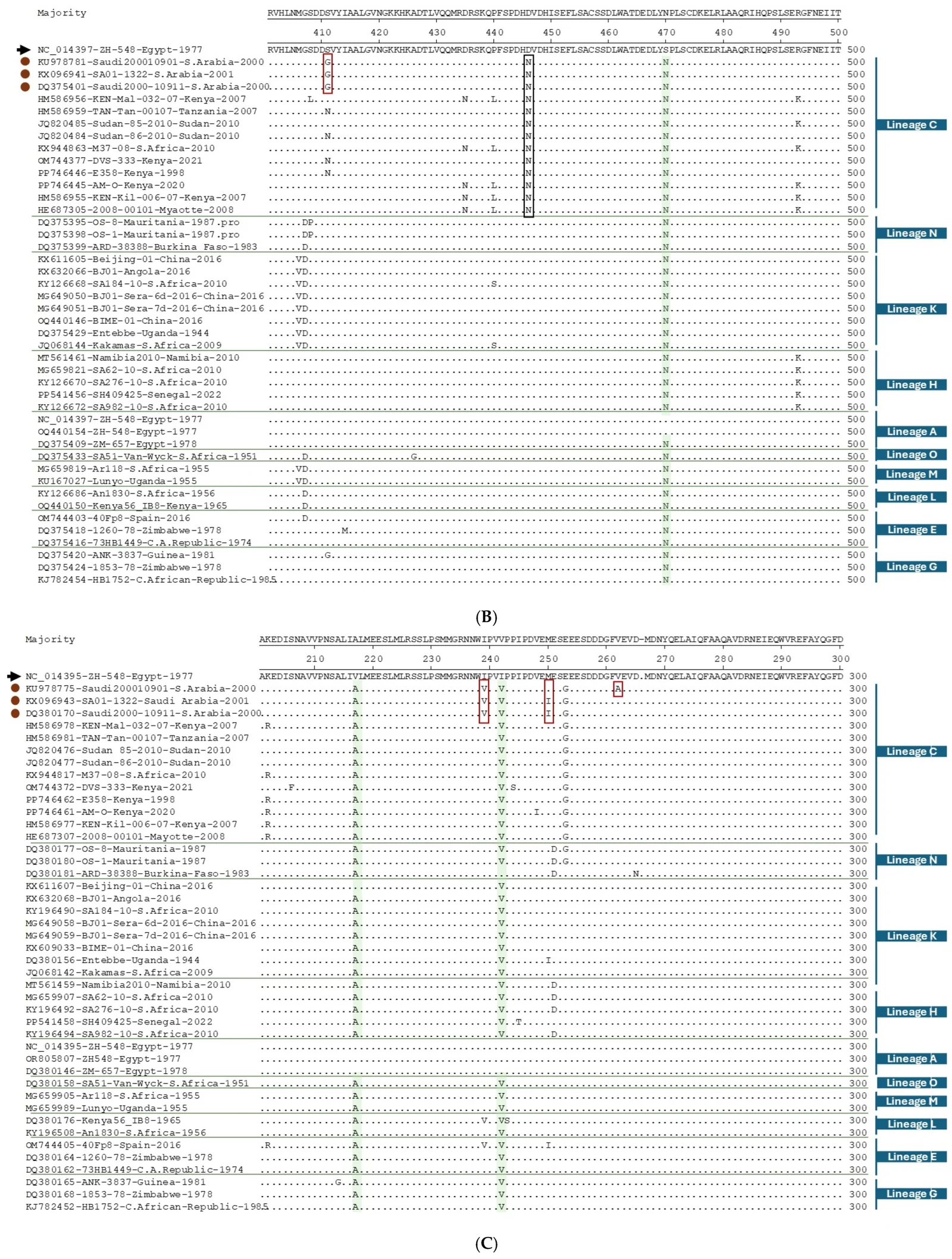
(**A**–**C**) Multiple sequence alignment of the complete protein sequences encoded by the three genomic segments of RVFV from Saudi Arabian isolates and representative international reference strains. The alignment was performed via the ClustalW method within the MegAlign program. A prototype/reference strain served as the reference sequence (indicated by arrow). Dots (.) represent residues identical to the reference. Major lineages are indicated on the right. Saudi Arabian isolates are highlighted by brown circles on the left. Lineage-specific amino acids are highlighted by rectangles. Unique amino acids for each strain (including Saudi-specific substitutions) are shown in red boxes, and permanent amino acid changes are shown in green rectangles. (**A**) M-segment-encoded polyprotein (aa: 600–700). (**B**) L-segment-encoded polymerase (aa: 400–500). (**C**) S-segment-encoded proteins (aa: 200–300). The remaining amino acid analysis across the full-length segments are shown in the Supplementary Figures (Figures S1–S36).

**Figure 3:**
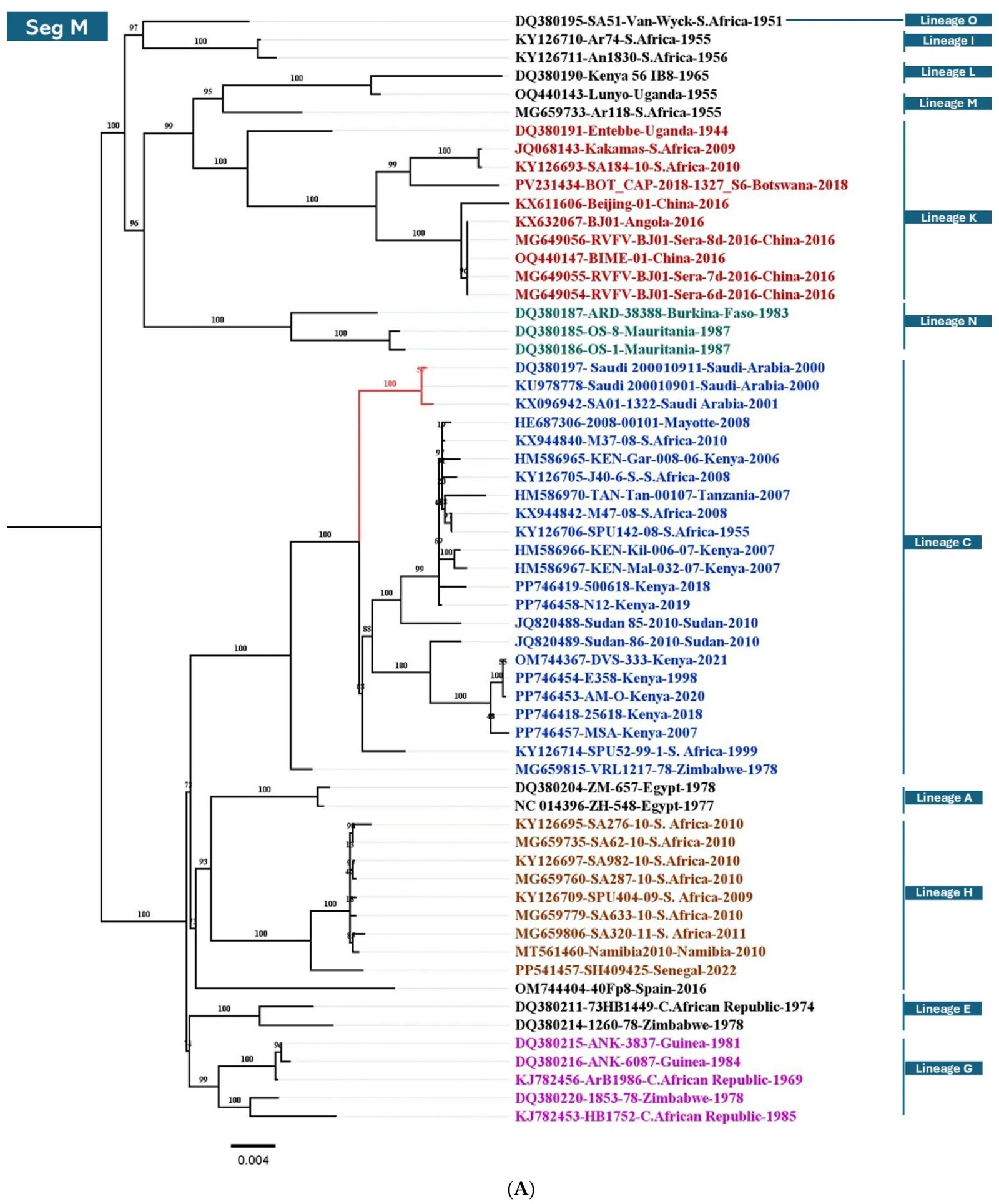

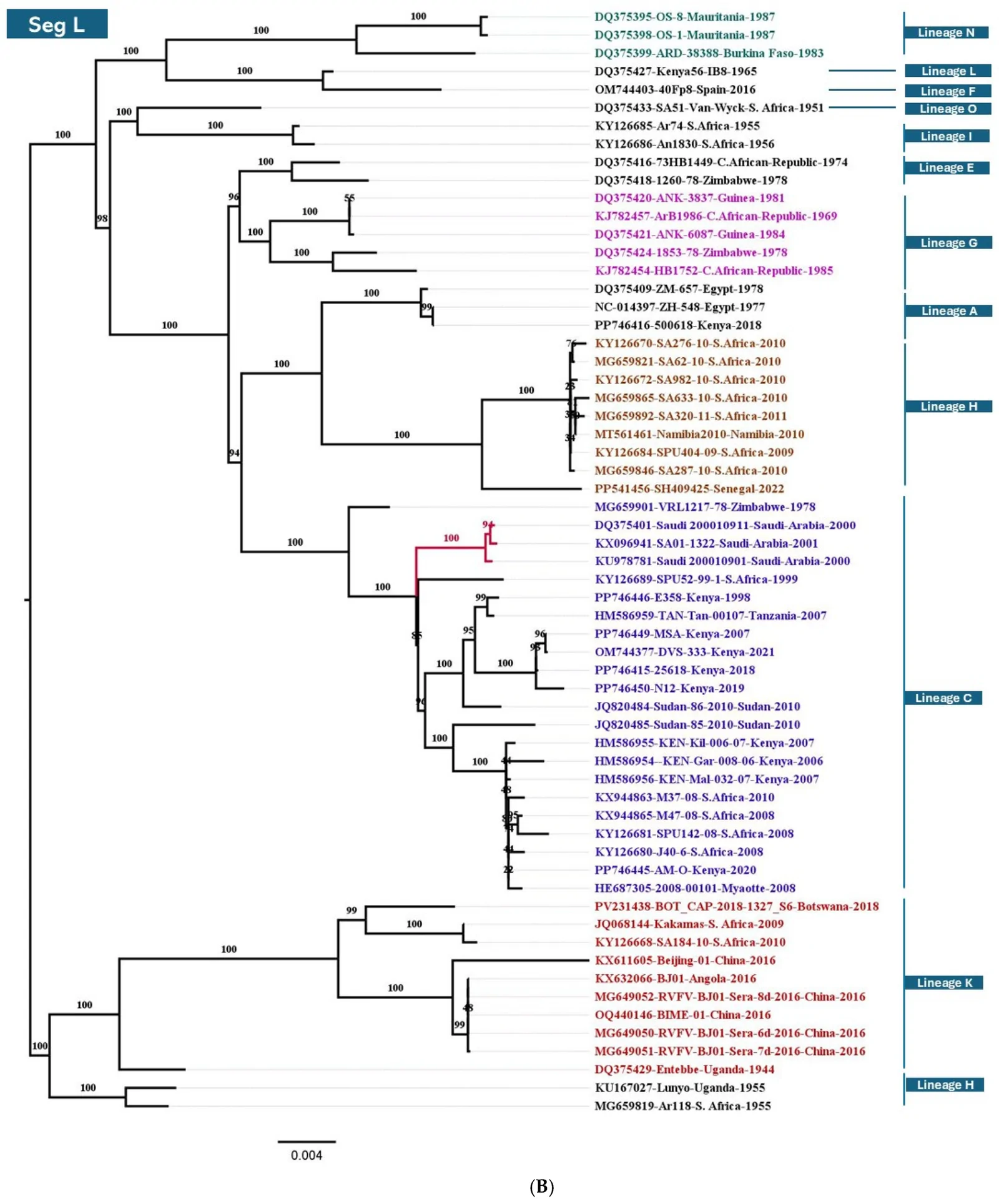

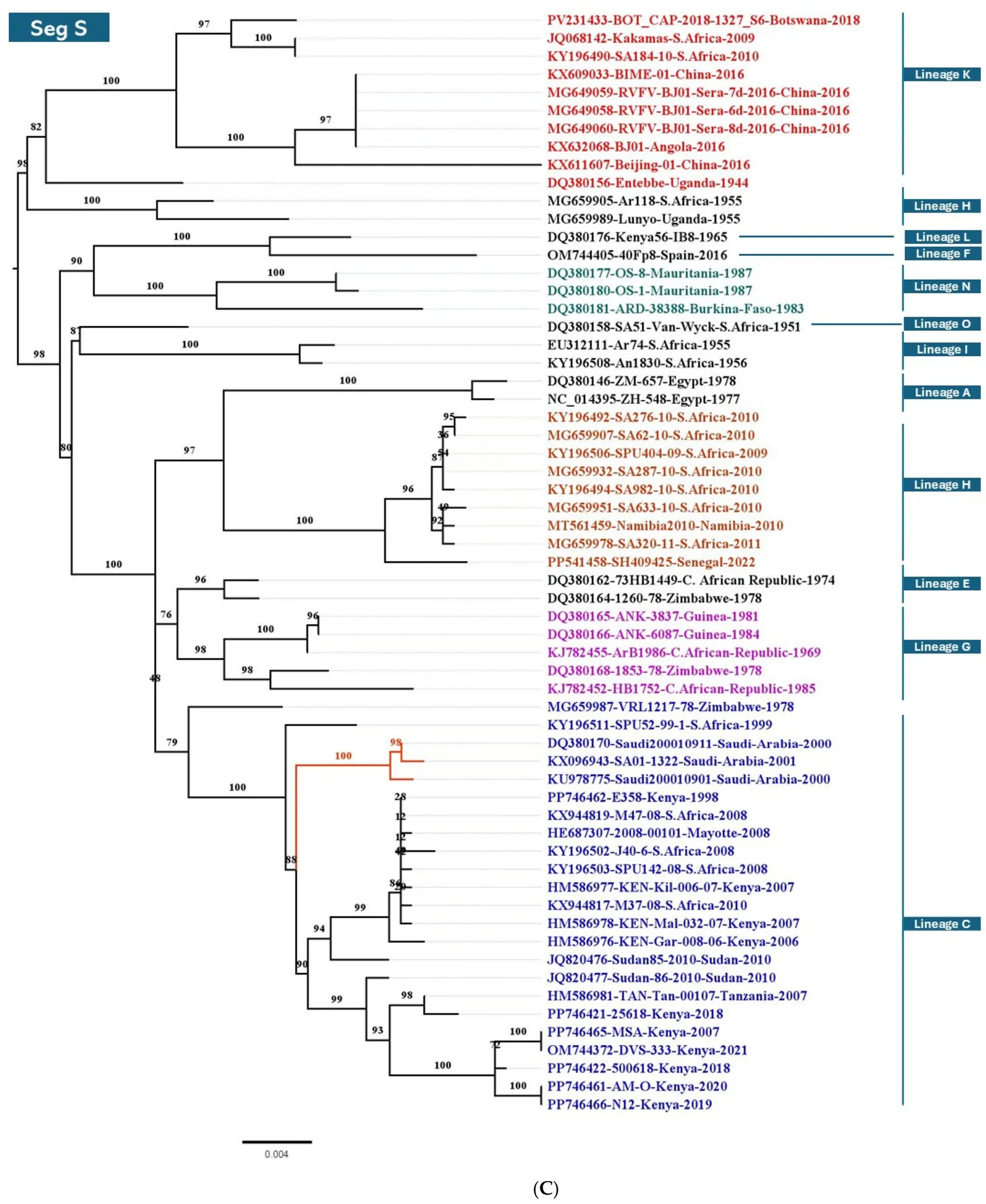
(**A**–**C**) Maximum-likelihood phylogenetic trees of the three genomic segments of RVFV reconstructed from a unified dataset of 61 complete genome sequences, including the three Saudi Arabian isolates and reference strains representing all major lineages (A–O). The best-fit nucleotide substitution model (GTR+I+G4) was selected for each segment using ModelFinder in IQ-TREE. Trees were inferred with IQ-TREE version 3.1.3 under the GTR+I+G4 model, applying 1000 ultrafast bootstrap replicates (UFBoot2) and 1000 SH-aLRT branch tests. Trees were rooted at the midpoint, visualized, and annotated in FigTree version 1.4.4. Bootstrap support values ≥ 70% are shown at the nodes. Scale bars indicate nucleotide substitutions per site. Major lineages are indicated on the right. Saudi strains form a well-supported monophyletic clade within Lineage C (bootstrap = 100) across all three trees and cluster most closely with East African strains from Kenya (2006–2007), Tanzania (2007), and South Africa (2008–2010). (**A**) M-segment tree (primary analysis used for lineage assignment). (**B**) L-segment tree. (**C**) S-segment tree. The congruent topologies across the L, M, and S segments provide no evidence of reassortment among the Saudi strains.

**Figure 4:**
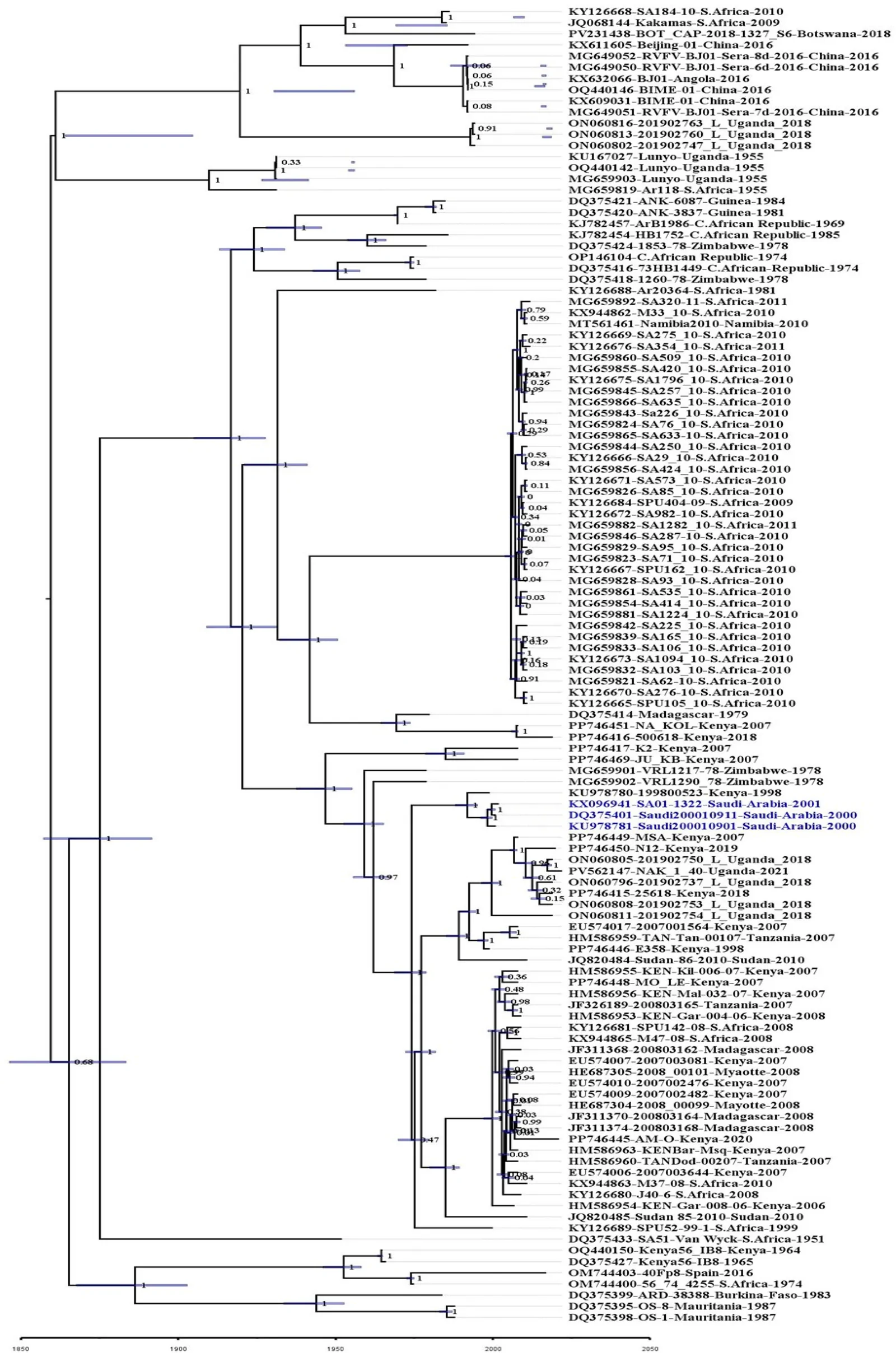
Maximum clade credibility (MCC) tree of 118 L-segment sequences (6279 nt) of RVFV inferred in BEAST under a GTR substitution model, strict molecular clock and constant-size coalescent prior. Node labels indicate posterior probabilities. The time scale is shown in calendar years. The three Saudi strains isolated during the 2000–2001 outbreak (DQ375401, KU978781 and KX096941) form a well-supported monophyletic cluster nested within a broader lineage that also includes sequences from Kenya, China and Angola.

**Table 1:** Summary of sequence datasets used in the present study.

Dataset	Genomic Segment(s)	Number of Sequences	Sampling Period	Analytical Purpose
Unified whole-genome dataset	L, M and S (complete)	61	1944–2022	Nucleotide and amino-acid variability analyses; maximum-likelihood phylogenetic reconstruction and lineage assignment; comparative topology assessment for reassortment; in silico N- and O-glycosylation prediction (M segment); selection-pressure analyses
Expanded L-segment dataset	L only	118	1951–2021	Bayesian molecular dating, and substitution-rate estimation.

**Table 2:** Summary of selection-pressure analyses performed on the complete coding sequences of RVFV L, M and S segments.

Segment	Method	Sites Under Purifying Selection ^a^	Sites Under Pervasive Positive Selection ^b^	Candidate Sites Under Episodic Diversifying Selection (MEME Only, *p* ≤ 0.05)	Codons Identified by MEME (Candidates)
L (RdRp)	FUBAR	1 050 (Prob ≤ 0.1)	0 (Prob ≥ 0.9)	–	–
	FEL	344 (*p* ≤ 0.05)	0 (*p* ≤ 0.05)	–	–
	SLAC	30 (P[dN/dS < 1] ≤ 0.05)	0 (P[dN/dS > 1] ≤ 0.05)	–	–
	MEME	–	–	6	239, 789, 1244, 1245, 1630, 1728
M (Gn/Gc)	FUBAR	581 (Prob ≤ 0.1)	0 (Prob ≥ 0.9)	–	–
	FEL	21 (*p* ≤ 0.05)	0 (*p* ≤ 0.05)	–	–
	SLAC	76 (P[dN/dS < 1] ≤ 0.05)	0 (P[dN/dS > 1] ≤ 0.05)	–	–
	MEME	–	–	7	173, 238, 286, 431, 554, 574, 631
S (N/NSs)	FUBAR	161 (Prob ≤ 0.1)	0 (Prob ≥ 0.9)	–	–
	FEL	82 (*p* ≤ 0.05)	0 (*p* ≤ 0.05)	–	–
	SLAC	30 (P[dN/dS < 1] ≤ 0.05)	0 (P[dN/dS > 1] ≤ 0.05)	–	–
	MEME	–	–	1	133

^a^ FUBAR: posterior probability of positive selection ≤ 0.1; FEL: *p*-value ≤ 0.05 for α > β; SLAC: binomial P[dN/dS < 1] ≤ 0.05. ^b^ FUBAR: posterior probability of positive selection ≥ 0.9; FEL: *p*-value ≤ 0.05 for β > α; SLAC: binomial P[dN/dS > 1] ≤ 0.05.

## Data Availability

The original contributions presented in the study are included in the article, further inquiries can be directed to the corresponding author.

## Supplementary Materials

The following supporting information can be downloaded at: https://www.mdpi.com/article/10.3390/v18101067/s1. Supplementary Figures provide additional figures of multiple sequence alignment of the complete of the 3 segments M (Figures S1–S11), L (Figures S12–S31), and S (Figures S32–S36) protein sequences of RVFV. Supplementary Tables provide accession numbers for the L, M, and S genome segments of all RVFV isolates used in this study, including host, geographic origin, and collection date (Table S1), List of RVFV L-segment sequences (accession, strain, country, and isolation year) employed in the divergence time analysis (Table S2), and Predicted N-linked and O-linked glycosylation sites on the RVFV 3 segments (Table S3). The selection pressure analysis folder contains files generated by the Datamonkey server.

## Abbreviations

The following abbreviations are used in this manuscript:

aa	Amino acid(s)
BIC	Bayesian Information Criterion
BSSVS	Bayesian Stochastic Search Variable Selection
ESS	Effective Sample Size
FEL	Fixed Effects Likelihood
FUBAR	Fast Unconstrained Bayesian AppRoximation
Gc	Glycoprotein C (G1)
Gn	Glycoprotein N (G2)
GTR	General Time Reversible
GTR+I+G4	General Time Reversible model with Invariant sites and Gamma distribution (4 categories)
HPD	Highest Posterior Density
IFN	Interferon
MCC	Maximum Clade Credibility
MCMC	Markov Chain Monte Carlo
MEME	Mixed Effects Model of Evolution
N	Nucleoprotein/Nucleocapsid protein
NCBI	National Center for Biotechnology Information
Ne	Effective population size
NSm	Non-structural protein M
NSs	Non-structural protein S
nt	Nucleotide(s)
RdRp	RNA-dependent RNA polymerase
RVF	Rift Valley fever
RVFV	Rift Valley fever virus
SH-aLRT	Shimodaira–Hasegawa approximate Likelihood Ratio Test
SLAC	Single-Likelihood Ancestor Counting
tMRCA	Time to the Most Recent Common Ancestor
UFBoot2	Ultrafast Bootstrap (version 2)
UTR	Untranslated region
WHO	World Health Organization
