# Supplementary material for: Evolutionary Dynamics of Rift Valley Fever Virus in Saudi Arabia: Whole-Genome Analysis of the 2000–2001 Outbreak Strains
Source: Viruses. 2026 Sep 26;18(10):1067. doi: 10.3390/v18101067 (PMC13709933; doi:10.3390/v18101067)
Supplement: Supplementary file 1 [file viruses-18-01067-s001.zip › Selection pressure analysis Files/Seg L/FEL/SegL-FEL.pdf]

FEL Analysis Visualization

**60**<br>sequences in the alignment	**2092**<br>codon sites in the alignment	**1**<br>partitions	**5**<br>median branches/partition used for testing
**1046**<br>non-invariant sites tested	**N/A**<br>parametric bootstrap replicates	**0**<br>sites under diversifying positive selection at $p \leq 0.1$	**383**<br>sites under purifying negative selection at $p \leq 0.1$
