# Supplementary material for: Evolutionary Dynamics of Rift Valley Fever Virus in Saudi Arabia: Whole-Genome Analysis of the 2000–2001 Outbreak Strains
Source: Viruses. 2026 Sep 26;18(10):1067. doi: 10.3390/v18101067 (PMC13709933; doi:10.3390/v18101067)
Supplement: Supplementary file 1 [file viruses-18-01067-s001.zip › Selection pressure analysis Files/Seg L/MEME/Seg L-MEME.pdf]

MEME Analysis Visualization

**60** sequences in the alignment	**2092** codon sites in the alignment	**1** partitions	**5** median branches/partition used for testing
**N/A** parametric bootstrap replicates	**10** sites subject to episodic diversifying selection	**1.10** median branches with support for selection/selected site	**0** sites with variable ω across branches
