# Supplementary material for: Evolutionary Dynamics of Rift Valley Fever Virus in Saudi Arabia: Whole-Genome Analysis of the 2000–2001 Outbreak Strains
Source: Viruses. 2026 Sep 26;18(10):1067. doi: 10.3390/v18101067 (PMC13709933; doi:10.3390/v18101067)
Supplement: Supplementary file 1 [file viruses-18-01067-s001.zip › Selection pressure analysis Files/Seg L/SLAC/Seg L-SLAC.pdf]

SLAC Analysis Visualization

SLAC Analysis Results

Single Likelihood Ancestor Counting (SLAC) uses a maximum likelihood approach to infer the number of synonymous and non-synonymous substitutions at each site.

18	2092	0	0
Sequences	Sites	Significant Sites	Positive Selection

0
Negative Selection
