# Supplementary material for: Evolutionary Dynamics of Rift Valley Fever Virus in Saudi Arabia: Whole-Genome Analysis of the 2000–2001 Outbreak Strains
Source: Viruses. 2026 Sep 26;18(10):1067. doi: 10.3390/v18101067 (PMC13709933; doi:10.3390/v18101067)
Supplement: Supplementary file 1 [file viruses-18-01067-s001.zip › Selection pressure analysis Files/Seg M/FEL/Seg M-FEL_analysis_2026-08.pdf]

FEL Analysis Visualization

**61** sequences in the alignment	**1197** codon sites in the alignment	**1** partitions	**5** median branches/partition used for testing
**669** non-invariant sites tested	**N/A** parametric bootstrap replicates	**0** sites under diversifying positive selection at $p \leq 0.1$	**177** sites under purifying negative selection at $p \leq 0.1$
