# Supplementary material for: Evolutionary Dynamics of Rift Valley Fever Virus in Saudi Arabia: Whole-Genome Analysis of the 2000–2001 Outbreak Strains
Source: Viruses. 2026 Sep 26;18(10):1067. doi: 10.3390/v18101067 (PMC13709933; doi:10.3390/v18101067)
Supplement: Supplementary file 1 [file viruses-18-01067-s001.zip › Selection pressure analysis Files/Seg M/MEME/Seg M- MEME.pdf]

MEME Analysis Visualization

**61**<br>sequences in the alignment	**1197**<br>codon sites in the alignment	**1**<br>partitions	**5**<br>median branches/ partition used for testing
**N/A**<br>parametric bootstrap replicates	**12**<br>sites subject to episodic diversifying selection	**1.25**<br>median branches with support for selection/ selected site	**0**<br>sites with variable ω across branches
