# Supplementary material for: Evolutionary Dynamics of Rift Valley Fever Virus in Saudi Arabia: Whole-Genome Analysis of the 2000–2001 Outbreak Strains
Source: Viruses. 2026 Sep 26;18(10):1067. doi: 10.3390/v18101067 (PMC13709933; doi:10.3390/v18101067)
Supplement: Supplementary file 1 [file viruses-18-01067-s001.zip › Supplementary Tables/Table S1-Accession numbers for the large (L), medium (M), and small (S) genome.pdf]

Table S1. Accession numbers for the large (L), medium (M), and small (S) genome segments of RVFV strains included in this study, with corresponding strain name, country of origin, and year of isolation.

No.	Seg M	Seg S	Seg L	Strain	Country	Year
1	DQ380187	DQ380181	DQ375399	ARD-38388	Burkina Faso	1983
2	DQ380190	DQ380176	DQ375427	Kenya 56 (IB8)	Kenya	1965
3	DQ380191	DQ380156	DQ375429	Entebbe	Uganda	1944
4	DQ380195	DQ380158	DQ375433	SA51-Van-Wyck	South Africa	1951
5	DQ380197	DQ380170	DQ375401	Saudi 2000-10911	Saudi Arabia	2000
6	KU978778	KU978775	KU978781	Saudi 200010901	Saudi Arabia	2000
7	KX096942	KX096943	KX096941	SA01-1322	Saudi Arabia	2001
8	DQ380214	DQ380164	DQ375418	1260-78	Zimbabwe	1978
9	DQ380215	DQ380165	DQ375420	ANK-3837	Guinea	1981
10	DQ380216	DQ380166	DQ375421	ANK-6087	Guinea	1984
11	DQ380220	DQ380168	DQ375424	1853-78	Zimbabwe	1978
12	HE687306	HE687307	HE687305	2008-00101	Mayotte	2008
13	HM586965	HM586976	HM586954	KEN-Gar-008-06	Kenya	2006
14	HM586966	HM586977	HM586955	KEN-Kil-006-07	Kenya	2007
15	HM586967	HM586978	HM586956	KEN-Mal-032-07	Kenya	2007
16	HM586970	HM586981	HM586959	TAN-Tan-00107	Tanzania	2007
17	JQ068143	JQ068142	JQ068144	Kakamas	South Africa	2009
18	JQ820488	JQ820476	JQ820485	Sudan 85-2010	Sudan	2010
19	JQ820489	JQ820477	JQ820484	Sudan 86-2010	Sudan	2010
20	KJ782453	KJ782452	KJ782454	HB1752	Central African Republic	1985

No.	Seg M	Seg S	Seg L	Strain	Country	Year
21	KJ782456	KJ782455	KJ782457	ArB1986	Central African Republic	1969
22	DQ380204	DQ380146	DQ375409	ZM-657	Egypt	1978
23	DQ380211	DQ380162	DQ375416	73HB1449	C. African Republic	1974
24	KX611606	KX611607	KX611605	Beijing-01	China	2016
25	KX632067	KX632068	KX632066	BJ01	Angola	2016
26	KX944840	KX944817	KX944863	M37-08	South Africa	2008
27	KX944842	KX944819	KX944865	M47-08	South Africa	2008
28	KY126693	KY196490	KY126668	SA184-10	South Africa	2010
29	KY126695	KY196492	KY126670	SA276-10	South Africa	2010
30	KY126697	KY196494	KY126672	SA982-10	South Africa	2010
31	KY126705	KY196502	KY126680	J40-6	South Africa	2008
32	KY126706	KY196503	KY126681	SPU142-08	South Africa	2008
33	KY126709	KY196506	KY126684	SPU404-09	South Africa	2009
34	KY126710	EU312111	KY126685	Ar74RSA55	South Africa	1955
35	KY126711	KY196508	KY126686	An1830	South Africa	1956
36	KY126714	KY196511	KY126689	SPU52-99-1	South Africa	1999
37	MG649054	MG649058	MG649050	RVFV-BJ01-Sera-6d-2016	China	2016
38	MG649055	MG649059	MG649051	RVFV-BJ01-Sera-7d-2016	China	2016
39	MG649056	MG649060	MG649052	RVFV-BJ01-Sera-8d-2016	China	2016
40	MG659733	MG659905	MG659819	Ar118	South Africa	1955

No.	Seg M	Seg S	Seg L	Strain	Country	Year
41	MG659735	MG659907	MG659821	SA62-10	South Africa	2010
42	MG659760	MG659932	MG659846	SA287-10	South Africa	2010
43	MG659779	MG659951	MG659865	SA633-10	South Africa	2010
44	MG659806	MG659978	MG659892	SA320-11	South Africa	2011
45	MG659815	MG659987	MG659901	VRL1217-78	Zimbabwe	1978
46	MT561460	MT561459	MT561461	Namibia2010	Namibia	2010
47	NC_014396	NC_01439 5	NC_014397	ZH-548	Egypt	1977
48	OM744367	OM744372	OM744377	DVS-333	Kenya	2021
49	OM744404	OM744405	OM744403	40Fp8	Spain	2016
50	OQ440143	MG659989	KU167027	Lunyo	Uganda	1955
51	OQ440147	KX609033	OQ440146	BIME-01	China	2016
52	PP541457	PP541458	PP541456	SH409425	Senegal	2022
53	PP746418	PP746421	PP746415	250618	Kenya	2018
54	PP746419	PP746422	PP746416	500618	Kenya	2018
55	PP746453	PP746461	PP746445	AM-O	Kenya	2020
56	PP746454	PP746462	PP746446	E358	Kenya	1998
57	PP746457	PP746465	PP746449	MSA	Kenya	2007
58	PP746458	PP746466	PP746450	N12	Kenya	2019
59	PV231434	PV231433	PV231438	BOT_CAP-2018-1327_S6	Botswana	2018
60	DQ380185	DQ380177	DQ375395	OS-8	Mauritania	1987
61	DQ380186	DQ380180	DQ375398	OS-1	Mauritania	1987
