# Supplementary material for: Evolutionary Dynamics of Rift Valley Fever Virus in Saudi Arabia: Whole-Genome Analysis of the 2000–2001 Outbreak Strains
Source: Viruses. 2026 Sep 26;18(10):1067. doi: 10.3390/v18101067 (PMC13709933; doi:10.3390/v18101067)
Supplement: Supplementary file 1 [file viruses-18-01067-s001.zip › Supplementary Tables/Table S2. List of RVFV L-segment seq divergence time analysis.pdf]

**Table S2.** List of RVFV L-segment sequences (accession, strain, country, and isolation year) employed in the divergence time analysis.

| No | Accession | Strain | Country | Year |
|---|---|---|---|---|
| 1 | KY126680 | J40-6 | South Africa | 2008 |
| 2 | DQ375401 | Saudi 2000-10911 | Saudi-Arabia | 2000 |
| 3 | KU978781 | Saudi200010901 | Saudi-Arabia | 2000 |
| 4 | KX096941 | SA01-1322 | Saudi Arabia | 2001 |
| 5 | KY126681 | SPU142-08 | South Africa | 2008 |
| 6 | KY126684 | SPU404-09 | South Africa | 2009 |
| 7 | KY126688 | Ar20364 | South Africa | 1981 |
| 8 | KY126689 | SPU52-99-1 | South Africa | 1999 |
| 9 | MG649050 | RVFV-BJ01-Sera-6d-2016 | China | 2016 |
| 10 | MG649051 | RVFV-BJ01-Sera-7d-2016 | China | 2016 |
| 11 | MG649052 | RVFV-BJ01-Sera-8d-2016 | China | 2016 |
| 12 | MG659819 | Ar118 | South Africa | 1955 |
| 13 | MG659821 | SA62-10 | South Africa | 2010 |
| 14 | MG659823 | SA71_10 | South Africa | 2010 |
| 15 | MG659824 | SA76_10 | South Africa | 2010 |
| 16 | MG659826 | SA85_10 | South Africa | 2010 |

| No | Accession | Strain | Country | Year |
|---|---|---|---|---|
| 17 | MG659828 | SA93_10 | South Africa | 2010 |
| 18 | MG659829 | SA95_10 | South Africa | 2010 |
| 19 | MG659832 | SA103_10 | South Africa | 2010 |
| 20 | MG659833 | SA106_10 | South Africa | 2010 |
| 21 | MG659839 | SA165_10 | South Africa | 2010 |
| 22 | MG659842 | SA225_10 | South Africa | 2010 |
| 23 | MG659843 | Sa226_10 | South Africa | 2010 |
| 24 | MG659844 | SA250_10 | South Africa | 2010 |
| 25 | MG659845 | SA257_10 | South Africa | 2010 |
| 26 | MG659846 | SA287-10 | South Africa | 2010 |
| 27 | MG659854 | SA414_10 | South Africa | 2010 |
| 28 | MG659855 | SA420_10 | South Africa | 2010 |
| 29 | MG659856 | SA424_10 | South Africa | 2010 |
| 30 | MG659860 | SA509_10 | South Africa | 2010 |
| 31 | MG659861 | SA535_10 | South Africa | 2010 |
| 32 | MG659865 | SA633-10 | South Africa | 2010 |
| 33 | MG659866 | SA635_10 | South Africa | 2010 |
| 34 | MG659881 | SA1224_10 | South Africa | 2010 |

| No | Accession | Strain | Country | Year |
|---|---|---|---|---|
| 35 | MG659882 | SA1282_10 | South Africa | 2011 |
| 36 | MG659892 | SA320-11 | South Africa | 2011 |
| 37 | MG659901 | VRL1217-78 | Zimbabwe | 1978 |
| 38 | MG659902 | VRL1290_78 | Zimbabwe | 1978 |
| 39 | MG659903 | Lunyo | Uganda | 1955 |
| 40 | MT561461 | Namibia2010 | Namibia | 2010 |
| 41 | OM744400 | 56_74_4255 | South Africa | 1974 |
| 42 | OM744403 | 40Fp8 | Spain | 2016 |
| 43 | ON060796 | 201902737_L | Uganda | 2018 |
| 44 | ON060802 | 201902747_L | Uganda | 2018 |
| 45 | ON060805 | 201902750_L | Uganda | 2018 |
| 46 | ON060808 | 201902753_L | Uganda | 2018 |
| 47 | ON060811 | 201902754_L | Uganda | 2018 |
| 48 | ON060813 | 201902760_L | Uganda | 2018 |
| 49 | ON060816 | 201902763_L | Uganda | 2018 |
| 50 | OP146104 | RSA/OBP/RVFVClone13/LAV | Central African Republic | 1974 |
| 51 | OQ440142 | Lunyo | Uganda | 1955 |

| No | Accession | Strain | Country | Year |
|---|---|---|---|---|
| 52 | OQ440146 | BIME-01 | China | 2016 |
| 53 | OQ440150 | Kenya56_IB8 | Kenya | 1964 |
| 54 | PP746415 | 25618 | Kenya | 2018 |
| 55 | PP746416 | 500618 | Kenya | 2018 |
| 56 | PP746417 | K2 | Kenya | 2007 |
| 57 | PP746445 | AM-O | Kenya | 2020 |
| 58 | PP746446 | E358 | Kenya | 1998 |
| 59 | PP746448 | MO_LE | Kenya | 2007 |
| 60 | PP746449 | MSA | Kenya | 2007 |
| 61 | PP746450 | N12 | Kenya | 2019 |
| 62 | PP746451 | NA_KOL | Kenya | 2007 |
| 63 | PP746469 | JU_KB | Kenya | 2007 |
| 64 | PV231438 | BOT-CAP-2018-1327_S6 | Botswana | 2018 |
| 65 | PV562147 | NAK_1_40 | Uganda | 2021 |
| 66 | DQ375395 | OS-8 | Mauritania | 1987 |
| 67 | DQ375398 | OS-1 | Mauritania | 1987 |
| 68 | DQ375399 | ARD-38388 | Burkina-Faso | 1983 |
| 69 | DQ375414 | MgH824 | Madagascar | 1979 |

| No | Accession | Strain | Country | Year |
|---|---|---|---|---|
| 70 | DQ375416 | 73HB1449 | Central African Republic | 1974 |
| 71 | DQ375418 | 1260-78 | Zimbabwe | 1978 |
| 72 | DQ375420 | ANK-3837 | Guinea | 1981 |
| 73 | DQ375421 | ANK-6087 | Guinea | 1984 |
| 74 | DQ375424 | 1853-78 | Zimbabwe | 1978 |
| 75 | DQ375427 | Kenya56-IB8 | Kenya | 1965 |
| 76 | DQ375433 | SA51-Van-Wyck | South Africa | 1951 |
| 77 | EU574006 | 2007003644 | Kenya | 2007 |
| 78 | EU574007 | 2007003081 | Kenya | 2007 |
| 79 | EU574009 | 2007002482 | Kenya | 2007 |
| 80 | EU574010 | 2007002476 | Kenya | 2007 |
| 81 | EU574017 | 2007001564 | Kenya | 2007 |
| 82 | HE687304 | 2008_00099 | Mayotte | 2008 |
| 83 | HE687305 | 2008_00101 | Mayotte | 2008 |
| 84 | HM586953 | KEN-Gar-004-06 | Kenya | 2008 |
| 85 | HM586954 | KEN-Gar-008-06 | Kenya | 2006 |
| 86 | HM586955 | KEN-Kil-006-07 | Kenya | 2007 |

| No | Accession | Strain | Country | Year |
|---|---|---|---|---|
| 87 | HM586956 | KEN-Mal-032-07 | Kenya | 2007 |
| 88 | HM586959 | TAN-Tan-00107 | Tanzania | 2007 |
| 89 | HM586960 | TANDod-00207 | Tanzania | 2007 |
| 90 | HM586963 | KENBar-Msq | Kenya | 2007 |
| 91 | JF311368 | 200803162 | Madagascar | 2008 |
| 92 | JF311370 | 200803164 | Madagascar | 2008 |
| 93 | JF311374 | 200803168 | Madagascar | 2008 |
| 94 | JF326189 | 200803165 | Tanzania | 2007 |
| 95 | JQ068144 | Kakamas | South Africa | 2009 |
| 96 | JQ820484 | Sudan-86-2010 | Sudan | 2010 |
| 97 | JQ820485 | Sudan85-2010 | Sudan | 2010 |
| 98 | KJ782454 | HB1752 | Central African Republic | 1985 |
| 99 | KJ782457 | ArB1986 | Central African Republic | 1969 |
| 100 | KU167027 | Lunyo | Uganda | 1955 |
| 101 | KU978780 | 199800523 | Kenya | 1998 |
| 102 | KX609031 | BIME-01 | China | 2016 |
| 103 | KX611605 | Beijing-01 | China | 2016 |

| No | Accession | Strain | Country | Year |
|---|---|---|---|---|
| 104 | KX632066 | BJ01 | Angola | 2016 |
| 105 | KX944862 | M33_10 | South Africa | 2010 |
| 106 | KX944863 | M37-08 | South Africa | 2010 |
| 107 | KX944865 | M47-08 | South Africa | 2008 |
| 108 | KY126665 | SPU105_10 | South Africa | 2010 |
| 109 | KY126666 | SA29_10 | South Africa | 2010 |
| 110 | KY126667 | SPU162_10 | South Africa | 2010 |
| 111 | KY126668 | SA184-10 | South Africa | 2010 |
| 112 | KY126669 | SA275_10 | South Africa | 2010 |
| 113 | KY126670 | SA276-10 | South Africa | 2010 |
| 114 | KY126671 | SA573_10 | South Africa | 2010 |
| 115 | KY126672 | SA982-10 | South Africa | 2010 |
| 116 | KY126673 | SA1094_10 | South Africa | 2010 |
| 117 | KY126675 | SA1796_10 | South Africa | 2010 |
| 118 | KY126676 | SA354_10 | South Africa | 2011 |
