# Supplementary material for: Evolutionary Dynamics of Rift Valley Fever Virus in Saudi Arabia: Whole-Genome Analysis of the 2000–2001 Outbreak Strains
Source: Viruses. 2026 Sep 26;18(10):1067. doi: 10.3390/v18101067 (PMC13709933; doi:10.3390/v18101067)
Supplement: Supplementary file 1 [file viruses-18-01067-s001.zip › Supplementary Tables/Table S3. Glycosylation profile tables.pdf]

Table S3. Predicted N-linked and O-linked glycosylation sites on the RVFV segment M protein.

Segment M (Net-O-Glycosylation sites)

Strain*	Positive Sites (amino acid positions)
DQ380185-OS-8-MAURITANIA-1987	400, 414, 797, 801, 806, 980, 984
DQ380186-OS-1-MAURITANIA-1987	400, 414, 797, 801, 806, 980, 984
DQ380190-KENYA56-IB8-1965	387, 414, 797, 801, 806, 980, 984
DQ380191-ENTEBBE-UGANDA-1944	414, 797, 801, 806, 980, 984
DQ380197-SA01-1322-S_ARABIA-2000	400, 414, 797, 801, 806, 980
DQ380204-ZM-657-EGYPT-1978	387, 414, 797, 801, 806, 980, 984
DQ380206-ZH-548-EGYPT-1977	387, 414, 797, 801, 806, 980, 984
DQ380211-73HB1449-C_A_REPUBLIC-1974	387, 414, 797, 801, 806, 980
DQ380214-1260-78-ZIMBABWE-1978	400, 414, 797, 801, 806, 980, 984
DQ380215-ANK-3837-GUINEA-1981	387, 414, 797, 801, 806, 980, 984
DQ380220-1853-78-ZIMBABWE-1978	387, 414, 797, 801, 806, 980, 984
DQ380187-ARD-38388-BURKINA-Faso-1983	387, 414, 797, 806, 980, 984
DQ380195-SA51-Burkina-Faso-1983	387, 414, 797, 801, 806, 980, 984
JQ068143-KAKAMAS-S.Africa-2009	387, 414, 797, 801, 806, 980, 984
JQ820488- Sudan 85-2010-Sudan-2010	387, 414, 797, 801, 806, 980
KX944840-M37-08- S.Africa-2008	400, 414, 797, 801, 806, 980, 984
KY126695-SA276-10- S.Africa-2010	400, 414, 797, 801, 806
HE687306-2008-00101-MAYOTTE-2008-M	387, 414, 797, 801, 806, 980, 984
HM586966-KEN-KIL-006-07-KENYA-2007	400, 414, 797, 801, 806, 980, 984
HM586967-KEN-MAL-032-07-KENYA-2007	178, 400, 414, 797, 801, 806, 980, 984
HM586970-TAN-TAN-00107-TANZANIA-2007	400, 414, 797, 801, 806, 980, 984

Strain*	Positive Sites (amino acid positions)
JQ820489-SUDAN-86-2010-SUDAN-2010	387, 414, 797, 801, 806, 980, 984
KJ782453-HB1752-C_AFRICAN-REPUBLIC-1985	387, 414, 797, 801, 806, 980, 984
KU978778-SAUDI-200010901-S_ARABIA-2000	400, 414, 797, 801, 806, 980
KX096942-SA01-1322-S_ARABIA-2001	387, 414, 797, 801, 806, 980
KX611606-BEIJING-01-CHINA-2016	178, 400, 414, 797, 801, 806, 980, 984
KX632067-BJ01-ANGOLA-2016	387, 414, 797, 801, 806, 980, 984
KY126693-SA184-10-S_AFRICA-2010	387, 414, 797, 801, 806, 980, 984
KY126697-SA982-10-S_AFRICA-2010	387, 414, 797, 801, 806
KY126711-AN1830-S_AFRICA-1956	414, 797, 801, 806, 980, 984
MG649054-BJ01-SERA-6D-2016-CHINA-2016	387, 414, 797, 801, 806, 980, 984
MG649055-BJ01-SERA-7D-2016-CHINA-2016	387, 414, 797, 801, 806, 980, 984
MG659733-AR118-S_AFRICA-1955	400, 414, 797, 801, 806, 980, 984
MG659735-SA62-10-S_AFRICA-2010	387, 414, 797, 801, 806
MT561460-NAMIBIA2010-NAMIBIA-2010	387, 414, 797, 801, 806
NC_014396-ZH-548-EGYPT-1977	387, 414, 797, 801, 806, 980, 984
OM744367-DVS-333-KENYA-2021	387, 414, 797, 801, 806, 980, 984
OM744404-40FP8-SPAIN-2016	400, 414, 797, 801, 806, 980, 984
OQ440143-LUNYO-UGANDA-1955	400, 414, 797, 801, 806, 980, 984
OQ440147-BIME-01-CHINA-2016	387, 414, 797, 801, 806, 980, 984
PP541457-SH409425-SENEGAL-2022	400, 414, 797, 801, 806, 980, 984
PP746453-AM-O-KENYA-2020_PRO	400, 414, 797, 801, 806, 980, 984
PP746454-E358-KENYA-1998	387, 414, 797, 801, 806, 980, 984

2. Segment M (Net-N-Glycosylation sites)

**Strain**	**Positive Sites (amino acid positions)**
NC_014396-ZH-548	88, 438, 794, 829, 1035, 1077
KU978778- SAUDI 200010901-S. Arabia-2000	88, 438, 794, 829, 1035, 1077
KX096942-SA01-1322-S-Arabia-2001	88, 438, 794, 829, 1035, 1077
DQ380197- Saudi 2000-10911-S-Arabia-2000	88, 438, 794, 829, 1035, 1077
HM586967-KEN-Mal-032-07-Kenya-2007	88, 438, 794, 829, 1035, 1077
HM586970-TAN-Tan-00107-Tanzania-2007	88, 438, 794, 829, 1035, 1077
JQ820488-Sudan-85-2010-Sudan-2010	88, 438, 794, 829, 1035, 1077
JQ820489-Sudan-86-2010-Sudan-2010	88, 438, 794, 829, 1035, 1077
KX944840-M37-08-S-Africa-2010	88, 438, 794, 829, 1035, 1077
PP746454-E358-Kenya-1998	88, 438, 794, 829, 1035, 1077
OM744367-DVS-333-Kenya-2021	88, 438, 794, 829, 1035, 1077
PP746453-AM-O-Kenya-2020	88, 438, 794, 829, 1035, 1077
HM586966-KEN-Kil-006-07-Kenya-2007	88, 438, 794, 829, 1035, 1077
HE687306-2008-00101-Mayotte-2008	88, 438, 794, 829, 1035, 1077
DQ380185-OS-8-Mauritania-1987	88, 438, 794, 829, 1077*
DQ380186-OS-1-Mauritania-1987	88, 438, 794, 829, 1035, 1077
DQ380187-ARD-38388-Burkina-Faso-1983	88, 438, 794, 829, 1035, 1077

Strain	Positive Sites (amino acid positions)
KJ782453-HB1752-C-African-Republic-1985	88, 438, 794, 829, 1035, 1077
KX611606-Beijing-01-China-2016	88, 438, 794, 829, 1035, 1077
KX632067-BJ01-Angola-2016	88, 438, 794, 829, 1035, 1077
KY126693-SA184-10-S-Africa-2010	88, 438, 794, 829, 1035, 1077
MG649054-BJ01-Sera-6d-2016-China-2016	88, 438, 794, 829, 1035, 1077
MG649055-BJ01-Sera-7d-2016-China-2016	88, 438, 794, 829, 1035, 1077
OQ440147-BIME-01-China-2016	88, 438, 794, 829, 1035, 1077
DQ380191-Entebbe-Uganda-1944	88, 438, 794, 829, 1035, 1077
JQ068143-Kakamas-S-Africa-2009	88, 438, 794, 829, 1035, 1077
MT561460-Namibia2010-Namibia-2010	88, 438, 794, 829, 1035, 1077
MG659735-SA62-10-S-Africa-2010	88, 438, 794, 829, 1035, 1077
KY126695-SA276-10-S-Africa-2010	88, 438, 794, 829, 1035, 1077
PP541457-SH409425-Senegal-2022	88, 438, 794, 829, 1035, 1077
KY126697-SA982-10-S-Africa-2010	88, 438, 794, 829, 1035, 1077
DQ380206-ZH-548-Egypt-1977	88, 438, 794, 829, 1035, 1077
DQ380204-ZM-657-Egypt-1978	88, 438, 794, 829, 1035, 1077
DQ380195-SA51- SA-51-Van-Wyck-1951	88, 438, 794, 829, 1035, 1077
MG659733-Ar118-S-Africa-1955	88, 438, 794, 829, 1035, 1077

Strain	Positive Sites (amino acid positions)
OQ440143-Lunyo-Uganda-1955	88, 438, 794, 829, 1035, 1077
KY126711-An1830-S-Africa-1956	88, 438, 794, 829, 1035, 1077
DQ380190-Kenya56-IB8-1965	88, 438, 794, 829, 1035, 1077
OM744404-40Fp8-Spain-2016	88, 438, 794, 829, 1035, 1077
DQ380214-1260-78-Zimbabwe-1978	88, 438, 794, 829, 1035, 1077
DQ380211-73HB1449-C-A-Republic-1974	88, 438, 517, 794, 829, 1035, 1077
DQ380215-ANK-3837-Guinea-1981	88, 438, 794, 829, 1035, 1077
DQ380220-1853-78-Zimbabwe-1978	88, 438, 794, 829, 1035, 1077

*Glycosylation predictions were performed on a subset of 43 representative sequences selected to cover all major RVFV lineages (A–O). This subset was chosen because the full-length sequences of some segments were too large, making analysis difficult.
